# Dynamics of Gene and Allelic Expression During Modern Hybrid Maize Breeding

**DOI:** 10.1111/pbi.70602

**Published:** 2026-02-26

**Authors:** Xuyang Liu, Yongxiang Li, Chunhui Li, Dengfeng Zhang, Guanhua He, Hui Liu, Shumin Sun, Yu Li, Mei Guo, Tianyu Wang

**Affiliations:** ^1^ State Key Laboratory of Crop Gene Resources and Breeding, Institute of Crop Sciences Chinese Academy of Agricultural Sciences Beijing China; ^2^ Beidahuang KenFeng Seed Co., Ltd Harbin China

**Keywords:** allele‐specific expression, breeding improvement, heterosis, maize, transcriptome

## Abstract

Maize breeding has greatly improved yield through single‐cross hybrids, but the underlying gene regulatory changes remain unclear. This study analysed transcriptomes of landmark maize hybrids and their parents across developmental stages and planting densities. Compared with their parents, hybrids showed a trade‐off in the expression of photosynthesis‐related genes and stress‐responsive genes. This expression rebalancing suggested a strategy that prioritises photosynthetic efficiency and growth vigour over stress defence mechanisms. Allele‐specific expression (ASE) analysis identified 19.9% of heterozygous loci exhibiting significant allelic imbalance, with notable enrichment in photosynthesis and stress response pathways. Importantly, the suppressed expression of deleterious alleles in hybrids not only correlated with phenotypic performance but also exhibited progressive enhancement through decades of breeding, indicating this regulatory mechanism has been selected during improvement. Consistent with this finding, breeding selection preferentially acted on *cis*‐regulatory regions, with stronger correlation between *cis*‐regulatory complementation of deleterious variants and hybrid release year compared to coding regions. Transcriptomic plasticity across environments was evaluated using the concept of entropy. Results showed that hybrids had lower transcriptomic entropy than their parental lines, and this reduction in entropy was significantly associated with heterosis. These findings highlight the critical role of allelic expression optimization in maize hybrid breeding and provide insights into the transcriptomic dynamics that underlie heterosis.

## 1 Introduction

Hybrid vigour, or heterosis, describes the enhanced phenotypic performance observed in hybrids relative to their parental lines (Hochholdinger and Baldauf 2018). This phenomenon has been exploited in agriculture since ancient times, with the earliest documented case being the breeding of hybrid kungas (donkey‐hemippe crosses) in Mesopotamia *ca*. 3000 bce for agricultural and military applications (Bennett et al. 2022). The scientific foundation of heterosis was established through Charles Darwin's systematic hybridization experiments in 1876, which first demonstrated its prevalence in plants (Darwin 1876). Maize ( *Zea mays* L.) has served as a model system for heterosis research and utilisation since the pioneering works in the early 1900s (East 1908; Shull 1908). Today, most maize cultivars are hybrids and account for over 35% of the total cereal production worldwide (Ter Steeg et al. 2022). Heterosis has not only enhanced crop performance but also driven the development of modern breeding systems, including critical technologies like male sterility (Wan et al. 2019).

Despite the extensive successes of heterosis utilisation in plant breeding, its genetic basis has long been debated (Birchler et al. 2006). Recent population genetic analyses revealed that dominant, overdominant and epistatic effects collectively contribute to heterosis (Huang et al. 2015, 2016; Jiang et al. 2017; Xiao et al. 2021). However, the underlying molecular mechanisms of heterosis still remain a mystery and not fully elucidated. To unlock the full potential of heterosis, it is important to develop comprehensive models beyond isolated genetic context (Birchler and Veitia 2007; Birchler et al. 2010). At the transcriptomic level, all possible gene expression modes, including additivity, dominance, underdominance, and overdominance, are widely distributed in hybrids of maize (Zhou et al. 2019; Li et al. 2021; Baldauf et al. 2022), rice (Shao et al. 2019) and *Arabidopsis* (Liu et al. 2021). These dominant or overdominant gene expression patterns are not directly linked to the classical genetic models, but the non‐additive gene expression could partially explain heterotic variance in hybrids (Pitz et al. 2025). Complementation of gene expression patterns in the hybrid could lead to hundreds of additionally expressed genes compared to either of the inbred parents (Li et al. 2021). This single parent expression (SPE) complementation was hypothesized to be advantageous for developmental plasticity and environmental adaptation in hybrids (Baldauf et al. 2022). Furthermore, the phenomenon that the alleles of the same gene are expressed unequally at the transcript level in hybrids is defined as allele‐specific expression (ASE) (Shao et al. 2019; Zhou et al. 2019), which may contribute to the phenotypic variations and underlie the genetic basis of heterosis (Hochholdinger and Baldauf 2018). While a major challenge in these studies is that these gene expression patterns have not been associated with heterotic phenotypes.

Over the past 60 years, the increase of maize production largely depended on the breeding of single‐cross hybrids. The genetic gain of desired traits and the underlying genetic or genomic changes during modern maize breeding have been characterised (Wang et al. 2020; Li et al. 2022). Improved biotic and abiotic stress tolerances, for instance adaptation to higher planting density, have been key contributors to yield increases (Duvick 2001). Furthermore, deleterious genetic variants play an important role in determining patterns of phenotypic variation and heterosis (Yang et al. 2017). The progressive purging of deleterious alleles and reduction of genetic load have been identified as key factors contributing to breeding success (Sun et al. 2023). However, despite these advances, our understanding remains incomplete regarding how allele‐specific regulation of deleterious variants in hybrids influences phenotypic variation and breeding progress.

This study presents a comprehensive transcriptomic analysis of landmark Chinese maize hybrids and their parental inbred lines in decades of breeding history. Our investigation reveals fundamental insights into the molecular basis of heterosis. First, we observed a trade‐off in gene expression patterns, with hybrids consistently upregulating photosynthesis‐related genes while downregulating stress‐responsive pathways compared to their parents. Second, allele‐specific expression analysis demonstrates that modern breeding has progressively enhanced the suppression of deleterious alleles, with this allelic expression pattern showing selection signatures during hybrid breeding. Finally, we established an association between heterosis and transcriptomic plasticity, as quantified by an entropy‐based metric of gene expression coordination across different conditions.

## 2 Results

### 2.1 Transcriptomic Profiling of Maize Hybrids and Parental Lines at Different Planting Densities and Developmental Stages

**FIGURE 1:**
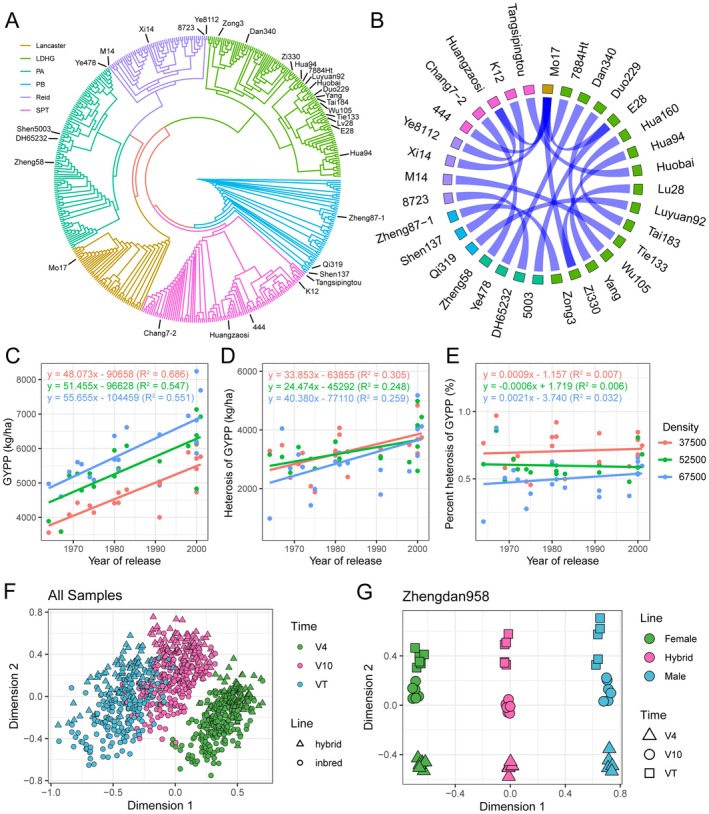
Pedigree and transcriptomic profiles of maize hybrids and parental lines. (A) The phylogenetic tree depicts the relationships among 450 representative inbred lines selected from our previous study of 1604 lines (Li et al. 2022). The clades of six heterotic groups are indicated with distinct colours. The 32 parental inbred lines utilised in the study are highlighted on the outer perimeter of the phylogenetic tree. (B) The Circos plot illustrates the crossing patterns of 20 hybrids. The colours of the boxes correspond to the different heterotic groups as indicated in (A). (C) The genetic gain of GYPP of the 20 hybrids used in this study under 37 500, 52 500 and 67 500 plants/ha. (D) The genetic gain of heterosis of GYPP under different planting densities. (E) The genetic gain of percent heterosis of GYPP under different planting densities. The phenotypic data is obtained from our previous study (Li et al. 2014). (F) The MDS plot displays transcriptomes of all RNA sequencing samples. The three developmental stages are highlighted by different colours, while hybrids and parental lines are distinguished by unique shapes. (G) The MDS plot focused on the hybrid Zhengdan958 along with its female parent Zheng58 and male parent Chang7‐2. The samples are differentiated based on their genotypes in dimension 1 and the three developmental stages in dimension 2.

To evaluate the gene expression dynamics underlying maize hybrid breeding, we conducted transcriptomic analysis of 20 historically significant single‐cross hybrids and the corresponding parental inbred lines (Table S1). These landmark hybrids and lines have dominated Chinese maize production (Li et al. 2014) and contributed foundational germplasm to modern breeding programs (Wang et al. 2023). The parental lines were categorised into six heterotic groups: Lancaster, LvDaHongGu (LDHG), PA, PB, Reid, and SiPingTou (SPT) (Figure 1A). The hybrids could be classified into 12 distinct cross patterns (Figure 1B). Phenotypic analysis revealed yield‐related traits, including grain yield per plot (GYPP), kernel weight per ear, kernel number per ear and biomass yield, exhibited highest levels of heterosis (Figure S1A). The genetic gain of GYPP was 48.073 to 55.655 kg/ha•year under different planting densities, in which heterosis contributed 24.474 to 40.380 kg/ha•year of GYPP (Figure 1C,D). The percent heterosis (relative to mid‐parent performance) did not increase significantly over the breeding period. This is largely attributable to the concurrent improvement in parental line performance. However, percent heterosis was consistently maintained at a high level (averaging 60.2% in landmark hybrids, Figure 1E). These results together demonstrate that modern breeding has achieved dual success: enhancing the performance of the parental lines and effectively harnessing heterosis to drive yield improvements.

To investigate the transcriptomic changes during this breeding process, the hybrids and parental inbred lines were grown under 120 000 plants/ha for high planting density (HPD) and 33 000 plants/ha for low planting density (LPD). We collected leaf samples at three developmental stages (V4: 4‐leaf vegetative stage; V10: 10‐leaf stage with reproductive initiation; VT: tasselling stage) for RNA sequencing. A total of 919 samples generated 6.96 Tb paired‐end Illumina sequencing data (Tables S2 and S3). Multidimensional scaling (MDS) and principal component analysis (PCA) revealed that transcriptomes clustered primarily by developmental stage, emphasising the significance of employing dynamic profiling approaches in gene expression analysis (Figure 1F, Figure S1B). When comparing the transcriptomes among each hybrid and its corresponding inbred lines, MDS and PCA clearly implied the pedigree relationships among the genotypes. Specifically, the transcriptomes of the hybrids consistently localised between their parental lines (Figure 1G; Figures S2 and S3).

### 2.2 Expression Trade‐Offs Between Photosynthesis and Stress Response Related Genes in Maize Hybrids and Parents

**FIGURE 2:**
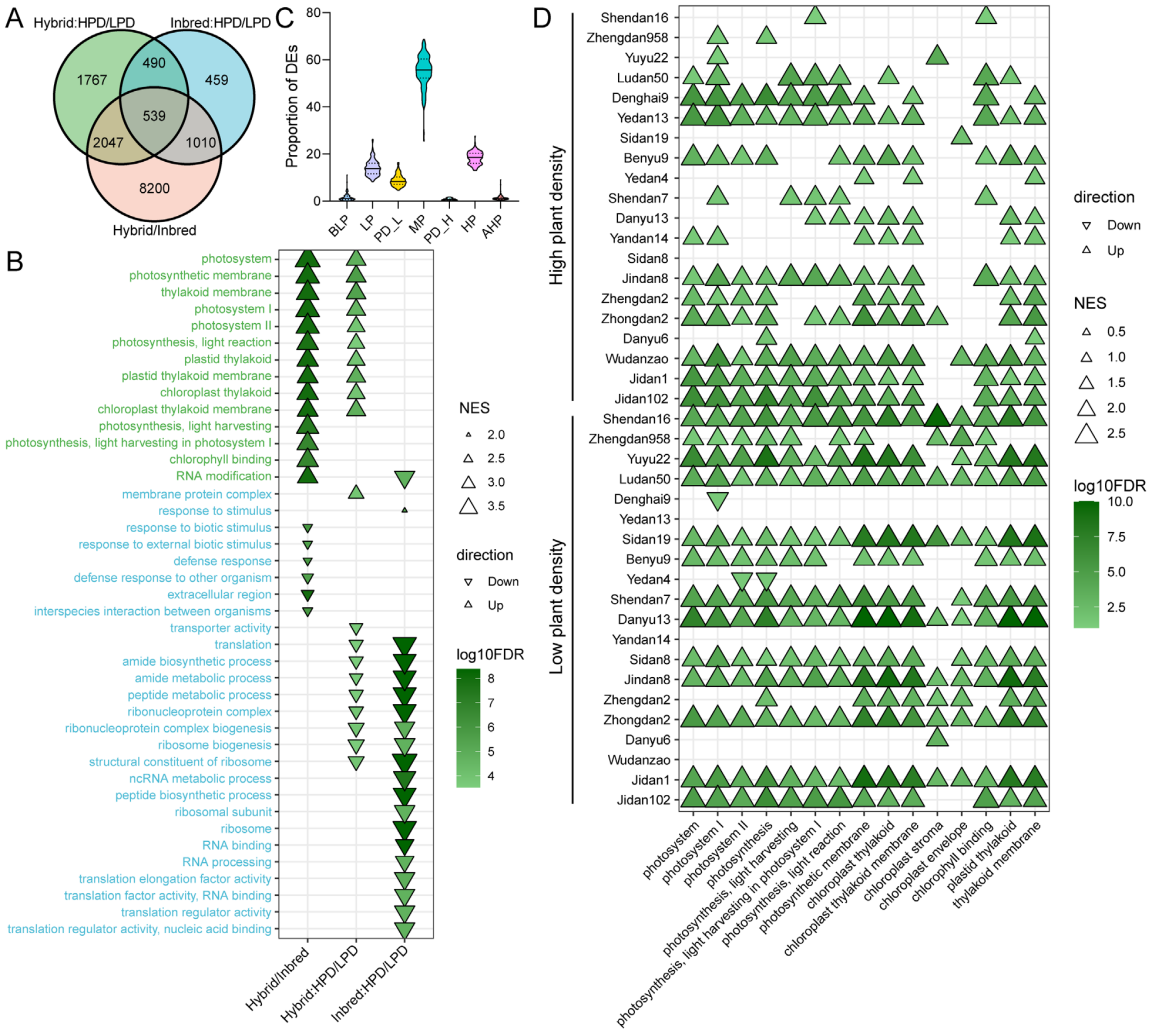
Gene expression changes between maize hybrids and parental lines. (A) Number of population‐level DE genes observed in different comparisons: Between hybrids and parental lines (Hybrid/Inbred), between HPD and LPD in hybrids (Hybrid:HPD/LPD), and between HPD and LPD in parental lines (Inbred:HPD/LPD). The DE analysis was performed using a multifactor model (see Methods) to estimate the effects of each factor and interaction on gene expression. (B) GO‐based GSEA of DE genes in Hybrid/Inbred, Hybrid:HPD/LPD and Inbred:HPD/LPD. The top 10 GO terms with the most significant enrichment are displayed. The direction of triangles indicates up‐regulation or down‐regulation in different comparisons. The size of triangles reflects normalised enrichment score (NES). The colour of triangles corresponds to log_2_FDR of GSEA. (C) Proportion of parental DE genes assigned to additive or non‐additive expression patterns in hybrids. The categories include mid‐parent expression (MP), high parent dominance expression (HP), low parent dominance expression (LP), low parent partial dominance expression (PD_L), high parent partial dominance expression (PD_H), above high parent overdominance expression (AHP), and below low parent overdominance expression (BLP). (D) GO‐based GSEA on the log_2_(D/A) of genes in each hybrid and planting density. The interpretation of the direction, size, and colour of the triangles follows the same conventions as outlined in panel (B).

To characterise population‐level gene expression changes across hybrids and parental inbred lines under varying planting density treatments, we applied a multiple‐factor differentially expressed (DE) gene analysis. We identified 4843 DE genes in hybrids and 2498 DE genes in parental inbred lines when comparing HPD to LPD, using a significance threshold of 0.01 false discovery rate (FDR) (Figure 2A; Tables S4 and S5). Notably, a substantially higher number of DE genes (11 796) emerged between hybrids and parents (Table S6). To further explore the functional relevance of these DE genes, we conducted gene ontology (GO)‐based gene set enrichment analysis (GSEA). This identified 110, 69 and 97 significant GO categories for Hybrid/Inbred, Hybrid:HPD/LPD and Inbred:HPD/LPD comparisons, respectively (Table S7–S9). An interesting finding is that the DE genes between hybrids and parental lines were enriched for up‐regulation of photosynthesis‐related functional categories and down‐regulation of stress response‐related GO terms (Figure 2B). This pattern implies that a critical trade‐off between reproductive capacity and stress defence mechanisms underpins the superior vigour observed in hybrids.

We further investigated the expression patterns of 267 and 2375 genes with photosynthesis and stress response GO terms across developmental stages in hybrids and parental lines. The analysis revealed a consistent pattern of differential expression in these functional categories. Photosynthesis‐related genes were predominantly up‐regulated in hybrids compared to inbred parents, while stress response genes were biased toward down‐regulation (Figure S4A–D). This inverse regulation was observed across multiple developmental stages, with a substantial proportion of photosynthesis‐related differentially expressed genes (59.8%–78.6%) exhibiting up‐regulation and stress response genes (67.5%–70.2%) showing down‐regulation in hybrids. Among photosynthesis‐related genes, *ZmAPO1*—a homologue of *Arabidopsis APO1* (Amann et al. 2004), which is essential for the stable assembly of photosystem complexes—exhibited consistent up‐regulation in the Zhengdan958 compared to parents Zheng58 and Chang7‐2 under at least three planting density and developmental stage combinations (Figure S4E). Similarly, *ZmLHCB2* and *ZmLHCB10*, which encode light‐harvesting chlorophyll a/b‐binding proteins that critical for efficient energy transfer (Rong et al. 2025), were significantly up‐regulated in Zhengdan958 compared to both parents (Figure S4F,G). For stress response genes, *ZmWRKY82*, a regulator of drought and salt tolerance (Wang et al. 2025; Wu et al. 2025), and *ZmWRKY106*, which modulates ABA signalling and antioxidant activities under drought and heat stress (Wang et al. 2018), were down‐regulated in the hybrid Zhengdan958 under multiple conditions (Figure S4H,I). Additionally, *ZmTPS7*, encoding a trehalose‐6‐phosphate synthase involved in stress adaptation via SnRK1 signalling (Liu et al. 2025), showed reduced expression in the hybrid Zhengdan958 (Figure S4J). This inverse regulation pattern supports the hypothesis of a trade‐off between photosynthetic efficiency and stress response in hybrids.

Single‐parent expression (SPE), where genes are exclusively active in one parental line, is a hypothesized mechanism underlying heterosis (Baldauf et al. 2022). We investigated the expression complementation of SPE genes in the hybrids. Across all parental lines, we identified 14 127 expressed genes (FPKM > 1), 7405 silent genes (FPKM < 0.1) and 10 789 genes with genotype‐dependent expression (Figure S1C). Hybrids showed parallel trends, with 15 511 expressed, 8986 silent, and 7545 diversely expressed genes (Figure S1D). Critically, parental pairs harboured 979.9 SPE genes on average (ranging from 468 to 1307), with 56.9% (averaging 557.3 genes, with a range of 187 to 788) exhibiting complementary expression (CE) in hybrids. Additionally, only an average of 13.9 genes were uniquely expressed in the hybrids and absent in both parental lines. The CE of SPE genes enabled hybrids to express a greater number of genes compared to their parents, this phenomenon consistently observed across all genotypes (Figure S1E). Furthermore, the numbers of DE genes and SPE genes were positively correlated with the genetic distance of all inbred lines as well as parental inbred pairs for the hybrids (Figure S5A–D). Similarly, the number of CE genes in hybrids was also positively correlated with the genetic distance of corresponding parents (Figure S5E).

We next characterised additive and non‐additive gene expression patterns across hybrids and their parental lines. Density distribution analysis of the dominance/additivity (D/A) ratio demonstrated that most genes exhibited additive expression in hybrids (Figure S6). Quantitatively, mid‐parent additive expression accounted for 55.0% of parental DE genes on average (Figure 2C). Dominant expression accounted for 32.5% of the parental DE genes, comprising 18.3% high‐parent and 14.1% low‐parent dominance. Partial dominance represented 9.6% (0.73% high‐parent partial dominance and 8.9% low‐parent partial dominance), while overdominant expression pattern was relatively rare (1.4% above high‐parent and 1.6% below low‐parent). The proportions of additive and non‐additive expression genes exhibited similar distributions across different developmental stages, planting density treatments, and genotypes (Table S10). Importantly, GO based GSEA revealed photosynthesis‐related categories enrichment for high‐parent overdominant genes in 15/20 hybrids under LPD and 19/20 under HPD (Figure 2D). This result is consistent with the up‐regulation of photosynthesis‐related genes between hybrids and parental lines (Figure 2B).

### 2.3 Allele‐Specific Expression in Different Maize Hybrids and Planting Densities

**FIGURE 3:**
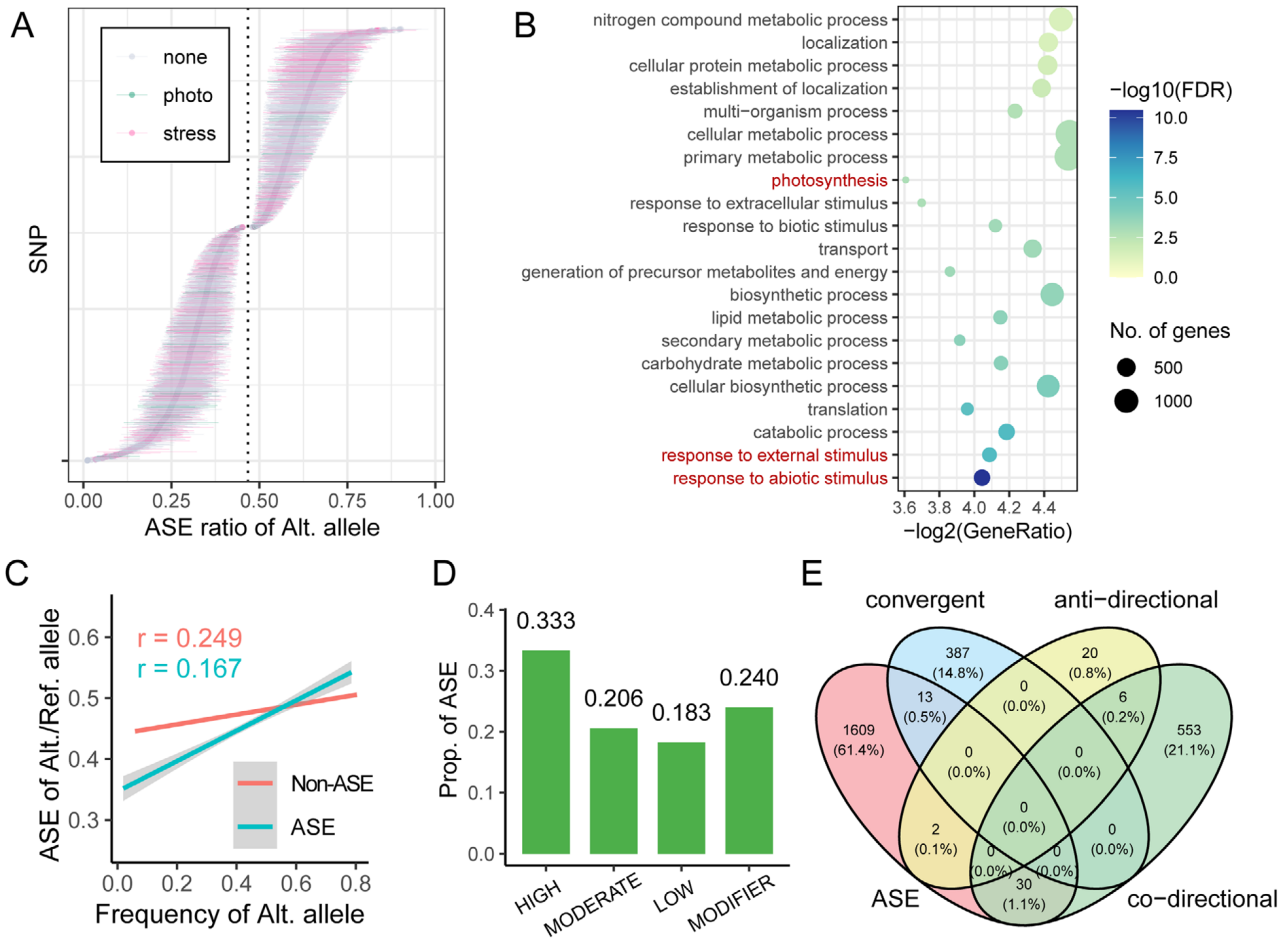
Allele‐specific expression of heterozygous variants in maize hybrids. (A) Estimates for 2857 SNPs exhibiting significant ASE at 0.05 FDR level. The dots are mean ASE ratio of alternative allele in hybrids and error bars indicate 97.5% credible intervals. The highlights in green are SNPs link to genes with function annotation of photosynthesis. The highlights in pink are SNPs link to genes with function annotation of stress response. The vertical dashed line indicates the median of ASE ratio for all analysed SNPs, which is set as the null hypothesis for Bayesian GLMM. Alt. allele: Alternative allele. (B) GO enrichment analysis of genes with significant ASE SNPs. The colour and size of dots indicate FDR and number of genes of corresponding GO terms. The GO terms related to stress response and photosynthesis are highlighted in red. (C) Correlation analysis between ASE ratio and frequency of alternative alleles that show significant ASE or non‐ASE. The Pearson correlation coefficient *r*_ASE_ and *r*_non‐ASE_ are 0.249 and 0.167, respectively. The *z*‐test *p* of these two correlations is 4.12 × 10^−5^. (D) Proportion of significant ASE SNPs with different predicted effect. All 14 345 SNPs used in ASE analysis were annotated by SnpEff. The predicted effect of variants classified into high, moderate, low and modifier categories. (E) Venn diagram shows the overlap of genes with significant ASE SNPs, co‐directional and anti‐directional selected genes between male and female heterotic groups, and convergent selected genes only in male heterotic groups (Li et al. 2022). The 30 ASE genes showed co‐directional selection and 13 ASE genes showed convergent selection are list in Table S12.

Allele‐specific expression (ASE) has emerged as an important regulatory mechanism contributing to phenotypic diversity and heterosis in hybrids (Shao et al. 2019). We employed a Bayesian generalised linear mixed model (GLMM) framework (McCoy et al. 2017) to comprehensively characterise ASE patterns across genetic backgrounds and environmental conditions. A total of 14 345 SNPs (covering 5699 genes) located in genic regions and heterozygous in at least 10 hybrids were used for ASE analysis. This approach identified 2857 significant ASE SNPs (19.9% of the analysed variants) within 1654 genes (29.0% of the covered genes) at FDR < 0.05, with effects modulated by both genotype and planting density (Figure 3A and Table S11).

Gene ontology analysis identified significant enrichment of stress‐responsive pathways among genes exhibiting ASE (Figure 3B), including several previously characterised stress tolerance related genes (Figure S7). The ASE SNP 1_52583301 (ASE ratio = 0.36, FDR = 3.6 × 10^−3^) locates in the 3′‐UTR of *ZmCIPK3*, which could enhance drought tolerance at seedling stage (Li et al. 2023). The SNP 1_10151181 (ASE ratio = 0.40, FDR = 7.2 × 10^−3^) lies within the CDS region of *ZmTIP1*, which is associated with survival rate under drought (Zhang et al. 2020). *ZmFER1* participates in resistance against *Fusarium* ear rot in maize (Liu et al. 2022), and SNP 4_186977877 in *ZmFER1* showed significant ASE (ASE ratio = 0.55, FDR = 6.2 × 10^−3^). In addition, the functional category of photosynthesis also showed significant enrichment in ASE genes. The SNP 2_10174140 (ASE ratio = 0.62, FDR = 6.7 × 10^−3^) is in *ZmAPO1*. *ZmLHCB7* (ASE ratio = 0.43, FDR = 9.1 × 10^−3^ of 6_169031288) and *ZmLHCB9* (ASE ratio = 0.22, FDR = 7.2 × 10^−3^ of 1_251755065) are the homologous genes of *AtLHCB7* in *Arabidopsis* and *PpLHCB9* in *Physcomitrella patens* , respectively. The loss‐of‐function mutation of *AtLHCB7* showed lower rates of photosynthesis (Peterson and Schultes 2014). The PpLHCB9 protein accumulated when grown in low light and recruited a large form of the photosystem I supercomplex (Pinnola et al. 2018).

Modern maize breeding has effectively reduced the frequency of deleterious variants, while these rare alleles correlate with dysregulation of expression and phenotypic variation (Kremling et al. 2018). Our analysis demonstrated that the ASE ratio of SNPs was positively correlated with the corresponding allele frequency, reflecting that alleles harbouring rare variants were lower expression in maize hybrids (Figure 3C). Comparative analysis revealed that SNPs with significant ASE showed higher allele frequency correlation (*r* = 0.249) than non‐ASE SNPs (*r* = 0.167), with this difference being statistically significant (*z*‐test *p* = 4.12 × 10^−5^). Low‐frequency variants (MAF < 0.1) showed a 3.6‐fold greater proportion of significant ASE (63.2%) than common variants (MAF > 0.1, 17.5%) (Figure S8A). To assess how variant deleteriousness affects allelic regulation, we classified SNPs by their predicted mutation effect using SnpEff annotation. Results showed that 33.3% of the SNPs predicted to have large mutation effect exhibited significant ASE, which is 1.6‐fold higher than that observed for SNPs with moderate, low, or modifier effects (Figure 3D).

A previous study has identified 1017 genes that display co‐directional or anti‐directional allele frequency changes in the female and male heterotic groups during modern breeding selection (Li et al. 2022). We compared the significant ASE genes to these 1017 co‐directional or anti‐directional genes and determined that the allele frequency of 30 ASE genes is changed codirectionally, 13 ASE genes are changed codirectionally between female and male heterotic groups, and 13 ASE genes are convergent changes only in the male heterotic group (Figure 3E and Table S12). Notably, these ASE genes include developmental regulators such as *RLD1*, an HD‐ZIP III transcription factor controlling leaf angle architecture (Juarez et al. 2004), and root development (Furutani et al. 2020), where we observed both significant ASE (ASE ratio = 0.40, FDR = 8.7 × 10^−3^) and co‐directional frequency changes in heterotic groups. *ZmMIPS1* exhibited significant ASE (ASE ratio = 0.27, FDR = 7.1 × 10^−3^) and showed convergent changes only in male heterotic groups, whose homologous gene *AtMIPS1* is essential for growth and flowering (Wang et al. 2024). These findings suggest that ASE genes are involved in the biological functions of growth and stress tolerance.

### 2.4 Allele‐Specific Response to Planting Density Stress in Maize Hybrids

**FIGURE 4:**
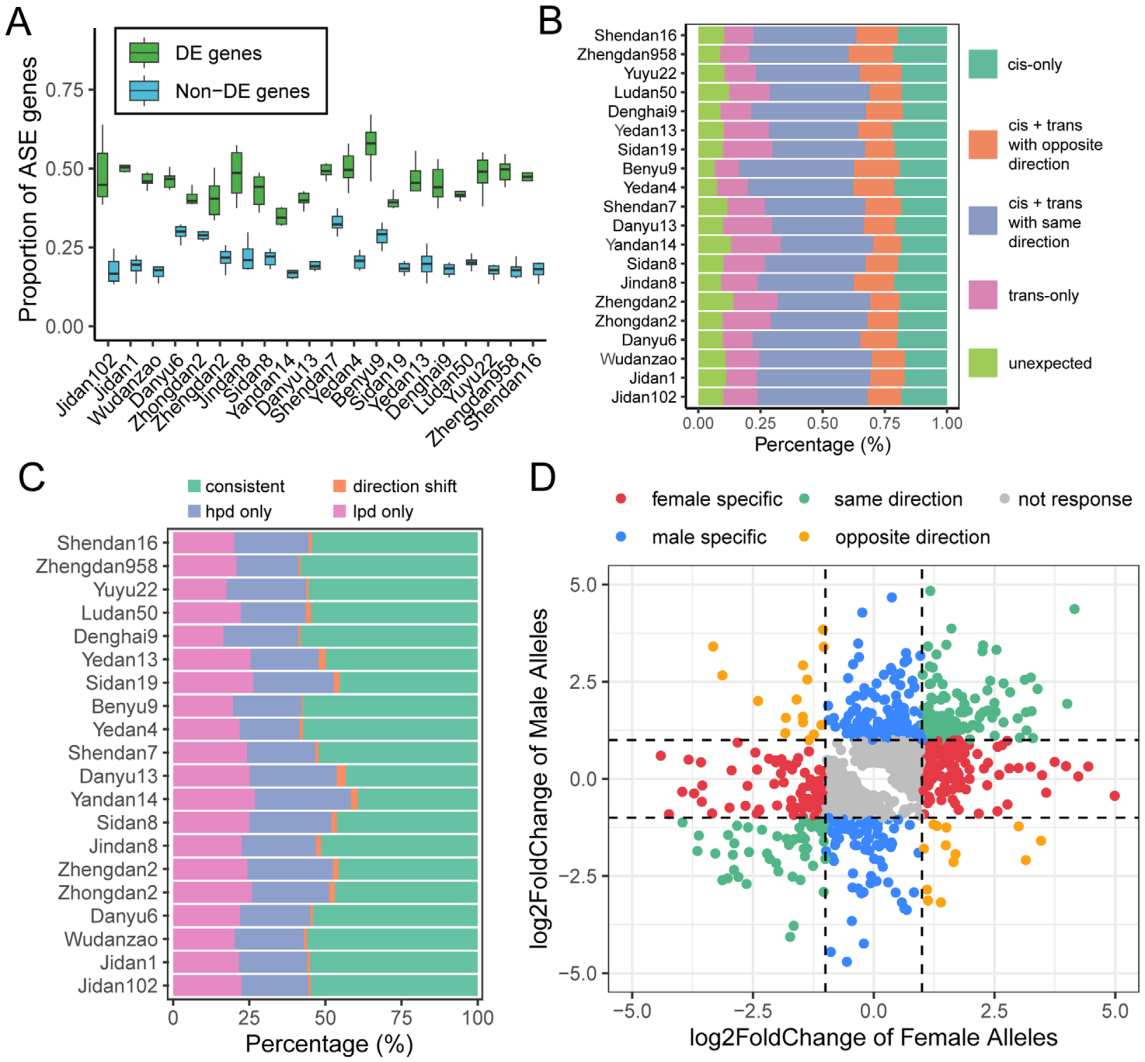
Haplotype based allele‐specific expression patterns in each maize hybrid. (A) Proportion of significant ASE genes across maize hybrids. ASE genes were classified based on parental expression profiles into parental DE genes and non‐DE genes. (B) Classification of ASE genes by regulatory pattern. “*trans*‐only” genes show equal allelic expression in hybrids despite differential expression in parents. “*cis*‐only” genes maintain the same allelic ratio as the parental fold change. “*cis* + *trans* (same direction)” genes have hybrid allelic ratios lower than parental DE fold change. “*cis* + *trans* (opposite direction)” genes show higher allelic ratios in hybrids than in parents. An additional unexpected pattern was identified where allelic ratios are opposite to parental DE direction. (C) ASE dynamics under planting density stress. Consistent ASE genes exhibit bias toward the same parental allele under both HPD and LPD. Direction‐shift ASE genes switch allelic bias between HPD and LPD. HPD‐only and LPD‐only ASE genes exhibit significant ASE under only one condition. (D) Allelic expression response to planting density stress. Expression of maternal and paternal alleles was quantified separately and tested for differential expression between HPD and LPD. Scatter plots show allele‐specific log_2_(fold changes); dashed lines indicate the log_2_(fold changes) thresholds of ±1. Significantly regulated alleles [log_2_(fold changes) > 1 or < −1 and FDR < 0.05] were categorised into: Same direction (both alleles up or down), opposite direction (one up, one down), and female‐ or male‐specific (only one parental allele shows response).

To further explore the ASE genes in each hybrid, we employed a haplotype phasing‐based pipeline (Castel et al. 2016) to quantify the maternal and paternal haplotype expression according to heterozygous variants at the gene level. Across hybrids, 22.4%–38.3% of analysed genes exhibited significant imbalanced expression (Binomial test FDR < 0.05) of parental alleles (Figure S8B). Comparative analysis between ASE in hybrids and DE in their parents demonstrated that parental DE genes had a higher probability of displaying ASE in hybrids (Figure 4A and Figure S9). And the proportion of ASE genes in hybrids increased with the enhanced fold change of DE genes in parents (Figure S8C). These allelic expression differences in hybrids suggest potential *cis*‐regulatory divergence. To test this, we examined variations in *cis*‐regulatory regions of genes with imbalanced and balanced allelic expression. Notably, genes with significant ASE harboured 1.14 to 1.37 times more genetic variants (SNPs and indels) in their *cis*‐regulatory regions than those with balanced expression (Figure S8D).

We subsequently applied a Bayesian information criterion model (Zhou et al. 2019) to elucidate the regulatory patterns underlying ASE in hybrids and DE in parents. Our analysis revealed that 19.4% of parental DE genes exhibited *cis*‐regulatory patterns in hybrids, where the allelic expression ratio is similar to the parental proportions (Figure 4B and Figure S10A). In contrast, 15.0% of parental DE genes displayed balanced allelic expression in hybrids, indicative of *trans*‐acting regulation. The majority (55.4%) showed combined *cis*‐ and *trans*‐regulatory effects, including 41.0% “*cis* + *trans* (same direction)” genes whose allelic ratios are lower than parental DE fold change and 14.4% “*cis* + *trans* (opposite direction)” genes that showed higher allelic ratios than parental DE. However, 10.2% of the parental DE genes were assigned into unexpected patterns, in which the allelic bias in the hybrids was in the completely opposite direction to that in the parents (Figure S11 and Table S13).

To investigate environmental influences on allele‐specific expression (ASE) in hybrids, we examined whether allelic expression bias could shift between planting density treatments. Remarkably, 51.8% of ASE genes maintained consistent parental expression bias under both LPD and HPD (Figure 4C and Figure S10B). While 22.6% and 24.2% of ASE genes showed uniquely ASE under LPD or HPD, respectively. Only 1.4% of ASE genes showed shifting ASE directions between LPD and HPD (Table S14).

We next examined how each parental allele responded to planting density stress in hybrid by analysing allele‐specific expression changes. Genes were assigned to different response patterns according to the expression fold change of female or male alleles between different planting density stresses (Figure 4D). The majority of stress‐responsive genes (66.4%) exhibited single‐allele responses, comprising nearly equal proportions of female (32.6%) and male (33.8%) allele‐specific responses. Coordinated responses were observed in 29.6% of genes where both alleles showed expression changes in the same direction. While a small subset of genes (4.0%) displayed antagonistic responses with opposite expression changes between parental alleles (Figure S12 and Table S15).

### 2.5 Change of Deleterious Allele Suppression During Maize Hybrid Breeding

**FIGURE 5:**
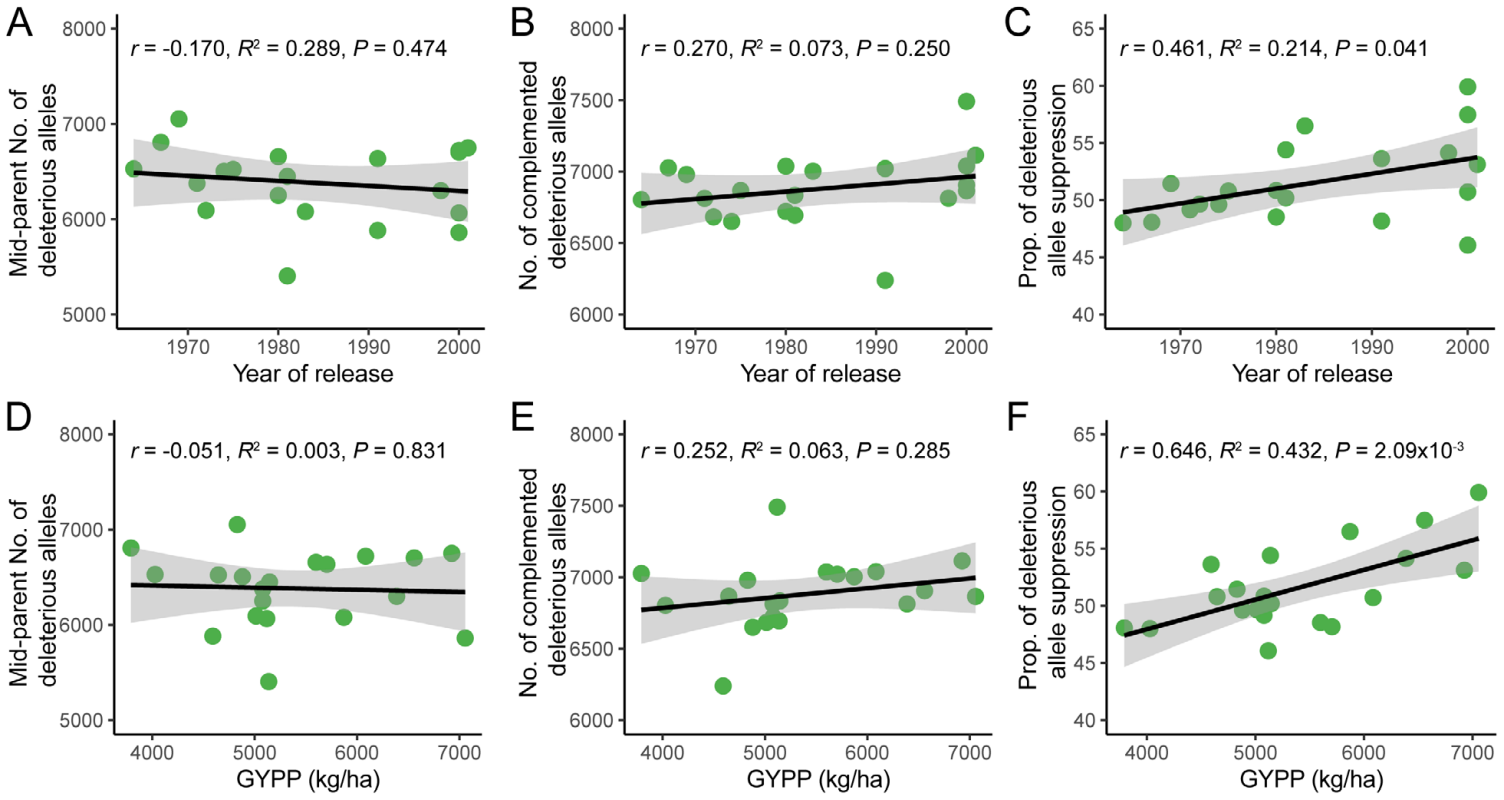
Changes of complementation and allelic expression of deleterious alleles during hybrid breeding. (A) Correlation between the mid‐parent number of deleterious alleles and the hybrid release year. The mid‐parent value is calculated as the average number of deleterious alleles from both parents. (B) Correlation between the number of complemented deleterious alleles in hybrids and their release year, indicating improvement through allelic complementation during breeding. (C) Correlation between the proportion of deleterious allele suppression and hybrid release year. (D–F) Correlation analysis of mid‐parent numbers of deleterious alleles, numbers of complemented deleterious alleles, and proportions of deleterious suppression toward GYPP of hybrids. The Pearson correlation coefficient *r*, *R* ^2^ of linear regression, and *p*‐value are presented.

The purging of deleterious variants represents a fundamental principle in the molecular basis underlying maize breeding improvement (Kremling et al. 2018). Complementation of the deleterious alleles plays an important role in the superior phenotypic performance of hybrids (Yang et al. 2017). We investigated changes in deleterious allele and expression patterns across hybrid maize lines subjected to decades of breeding selection. Our analysis revealed a decreasing trend in the total number of deleterious alleles in hybrids over breeding history (*r* = −0.17, Figure 5A). Conversely, we observed a positive association between hybrid release year and the number of complemented deleterious alleles (*r* = 0.27, Figure 5B), defined as genes carrying deleterious variants in one parental allele but not the other. Phenotypic analysis showed that grain yield tended to correlate positively with the number of complemented deleterious alleles (*r* = 0.25) and weakly negatively with total deleterious allele count (*r* = −0.051, Figure 5D,E). These results collectively suggest a breeding selection trend toward reduced deleterious allele load and increased heterozygosity at these loci.

Transcriptional suppression of deleterious variants may represent an important protective mechanism against their deleterious phenotypic effects (Rivas et al. 2015). Our analysis of complementary deleterious allele pairs revealed that 43.6%–62.6% of these pairs exhibited deleterious allele suppression, characterised by higher expression of functional alleles relative to their deleterious counterparts (Figure S10C). Notably, the proportion of suppressed deleterious alleles showed significant positive correlation with hybrid release year (*r* = 0.46, *p* = 0.04; Figure 5C), indicating that modern breeding selection not only eliminates or complements deleterious variants but also preferentially maintains their low expression levels. Importantly, this suppression phenomenon was positively associated with GYPP (*r* = 0.65, *p* = 2.09 × 10^−3^), suggesting its functional relevance to yield enhancement in elite hybrids (Figure 5F).

Our findings on deleterious allele suppression implicate *cis*‐regulatory mechanisms in improvement of hybrid. To evaluate whether *cis*‐regulatory regions experience stronger selection pressure than coding sequences during breeding, we analysed complemented deleterious variants using genomic evolutionary rate profiling (GERP) (Swarts et al. 2017). Comparative analysis revealed that the accumulation of complemented deleterious variants in *cis*‐regulatory regions had stronger correlation with hybrid releasing year (*r* = 0.15 for *cis*, *r* = −0.073 for CDS), GYPP (*r* = 0.18 for *cis*, *r* = −0.064 for CDS) and heterosis of GYPP (*r* = 0.38 for *cis*, *r* = 0.12 for CDS) than CDS regions (Figure S13). We further performed an eGWAS analysis and identified 3 572 171 *cis*‐eQTNs associated with 19 208 genes (Figure S14). These *cis*‐eQTNs were significantly (*p* < 0.001) enriched in the breeding selection signatures of different heterotic groups (Li et al. 2022; Figure S14). These results demonstrate preferential selection on *cis*‐regulatory elements during maize breeding improvement.

### 2.6 Heterosis Feeds on “Negative Entropy”

**FIGURE 6:**
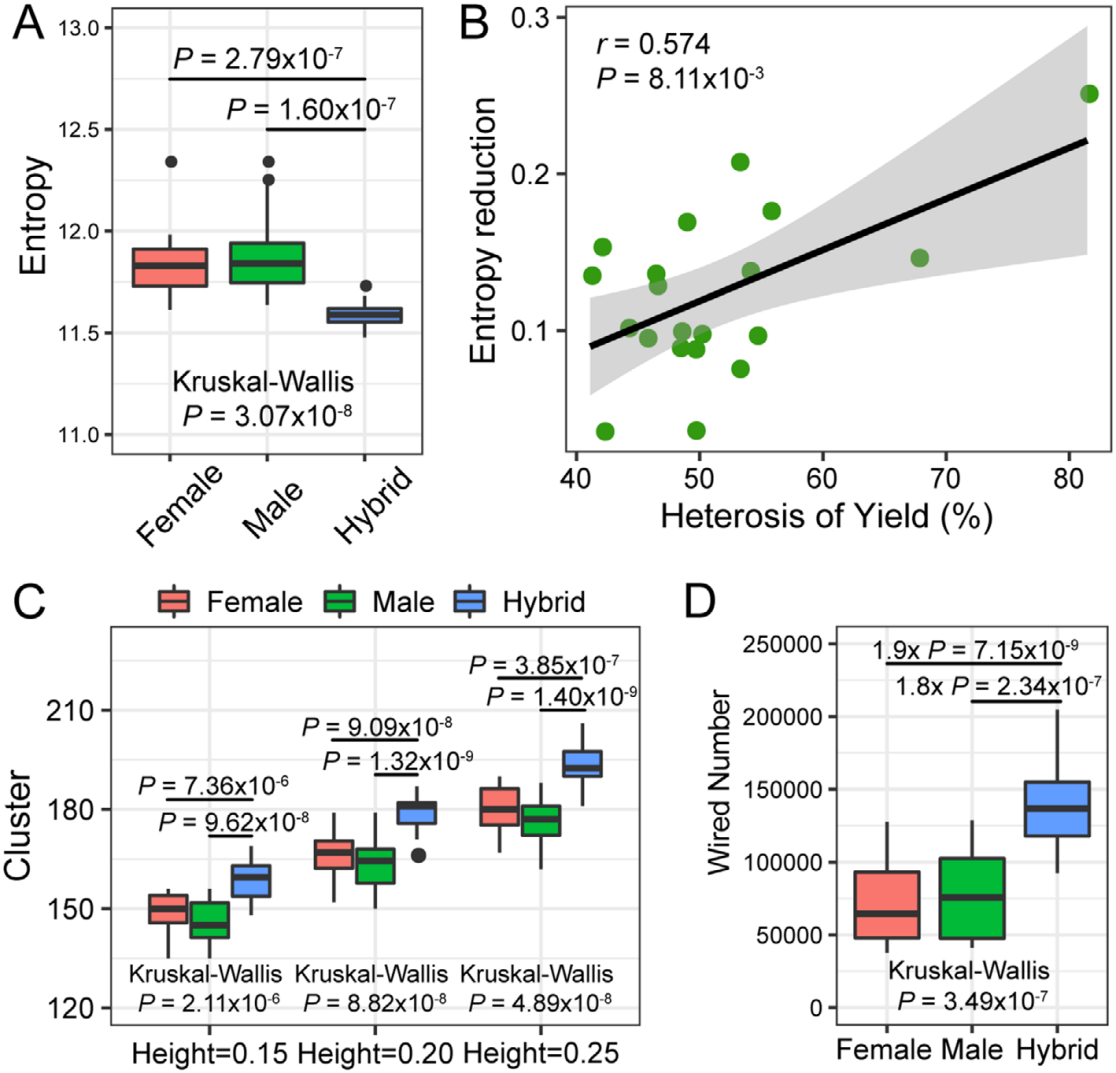
Transcriptomic plasticity of hybrids and parents. (A) The transcriptomic plasticity of each genotype was calculated using Shannon's entropy. The boxplot showed significantly reduced entropy in maize hybrids. Significance tests were performed using pairwise Student's *t*‐test and Kruskal‐Wallis test. (B) Correlation of transcriptomes entropy reduction with grain yield heterosis in maize hybrids. (C) Number of co‐expression clusters in maize hybrids and parents at different parameters. (D) Number of transcription factor‐target gene wires in gene regulatory networks of maize hybrids and parental inbreds.

We hypothesized that the superior fitness of the hybrid is relevant to the enhanced dynamics or plasticity of the gene regulation networks. Inspired by Schrödinger's concept of “negative entropy” as a fundamental life principle (Schrödinger 1944), we applied Shannon's entropy analysis to assess the transcriptomic dynamics. Maize hybrids exhibited significantly lower transcriptomic entropy than their parental inbred lines (Figure 6A and Figure S15). The observed reduction in Shannon's entropy within hybrid transcriptomes indicates a less uniform and more structured distribution of gene expression across developmental stages and planting densities. In the context of information theory, this lower entropy indicates a state of higher order and reduced randomness, which we interpret as a more coordinated and potentially more flexible transcriptomic configuration, enabling optimized responses to varying growth conditions. Importantly, the extent of entropy reduction correlated positively with heterosis levels for key agronomic traits, including GYPP, kernel weight, kernel number, and biomass yield (Figure 6B and Figure S16). This phenomenon extended beyond maize, as analysis of *Arabidopsis* hybrid (Col‐0 × Per‐1) similarly showed reduced transcriptomic entropy during seedling development compared to parental ecotypes, supporting conserved mechanisms of transcriptional plasticity in plant hybrids (Figure S17).

To verify the enhanced transcriptomic dynamics in hybrids, we conducted hierarchical clustering analysis of co‐expression networks across different planting densities and developmental stages. Hybrids exhibited significantly more co‐expression clusters than their parental lines (Figure 6C), indicating enhanced transcriptional flexibility. We further constructed gene regulatory networks (GRNs) to examine transcription factor‐target gene interactions. Comparative analysis revealed substantial network expansion in hybrids, with 1.52‐ and 1.49‐fold increases in connected genes relative to female and male parents, respectively (Figures S18A–E and S19). Network complexity was similarly increased, with 1.92‐ and 1.81‐fold more gene pairs than in either parent (Figures S18F and S20). Notably, 39.2% of hybrid GRN connections represented novel interactions absent in both parents (Figures S18G and S21). And only 2.88% of the wired gene pairs of GRNs in hybrids were inherited from their parents (Figures S18H and S22). These findings demonstrate that hybridization induces profound transcriptomic rewiring, suggesting network‐level mechanisms may complement genetic models in explaining heterosis (Birchler et al. 2010).

## 3 Discussion

Since the development of single‐cross hybrid in the 1960s, the yields of maize have increased 4.5‐fold in the United States and 2.7‐fold in China, the world's two largest maize producing countries (FAO 2023). Understanding the molecular basis of breeding improvement is of great importance to enhance breeding efficiency. Previous studies have investigated the genetic and genomic changes during maize domestication and improvement (Hufford et al. 2012; Liu et al. 2015; Wang et al. 2017, 2020; Li et al. 2022). Current evidence suggests that domestication bottlenecks increased deleterious allele frequencies, while modern breeding has progressively reduced this genetic load (Wang et al. 2017; Yang et al. 2017; Lozano et al. 2021). Furthermore, the accumulation of superior alleles contributes to phenotypic variation, which has been reported in large populations of rice (Huang et al. 2015, 2016) and maize (Liu et al. 2020). Our analysis of historically significant Chinese hybrids reveals a continuing decline in deleterious allele number during modern breeding. Notably, the proportion of complemented deleterious alleles positively correlated with the release year and grain yield of hybrids (Figure 5). These findings demonstrate that complementation of the deleterious alleles has been under selection and contributed to modern hybrid maize breeding.

**FIGURE 7:**
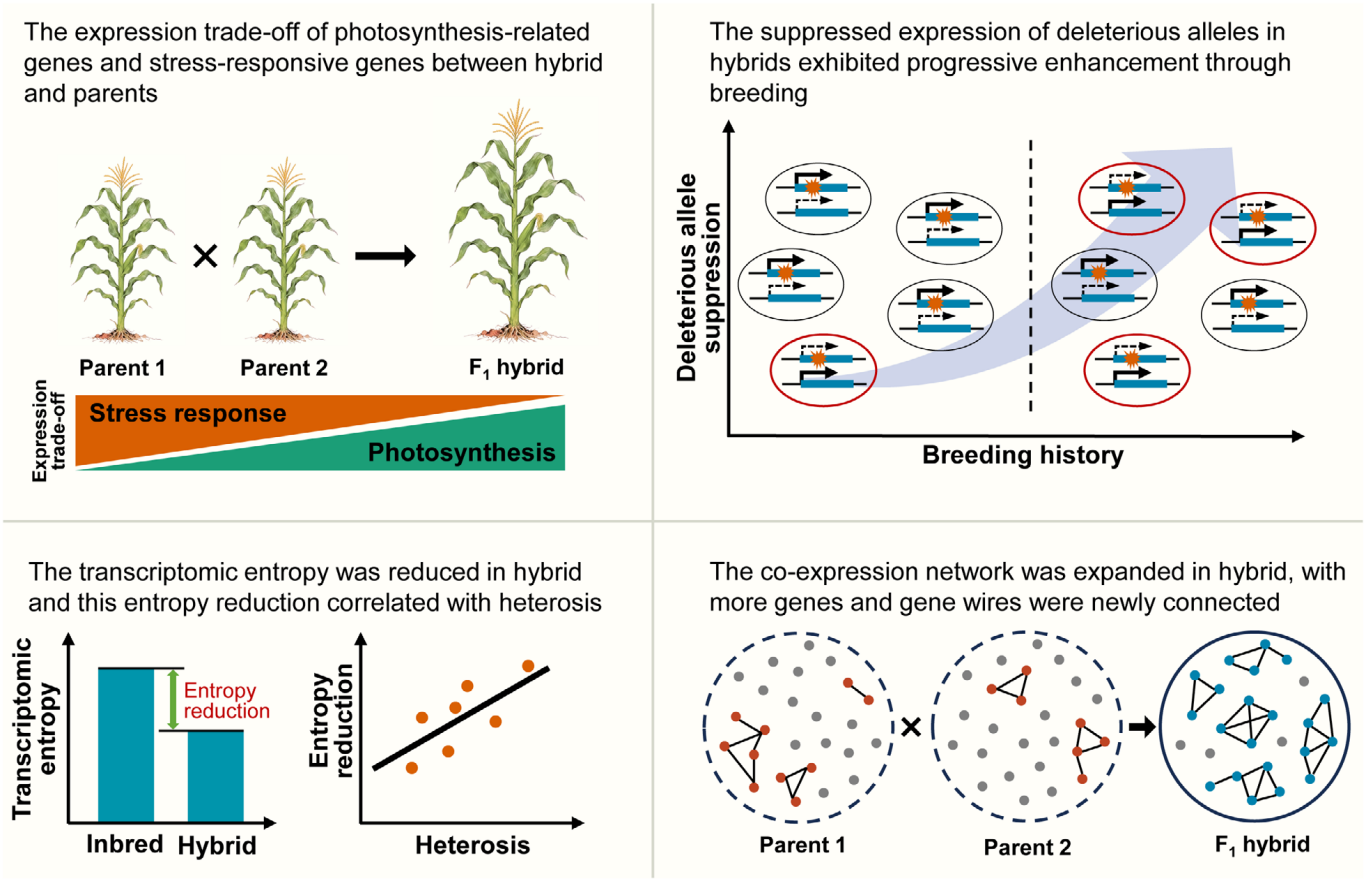
Graphical summary of the major findings in the present study. First, hybrids exhibit upregulated photosynthesis‐related genes and downregulated stress‐responsive genes, optimising growth vigour over stress defence. Second, breeding progressively enhances suppression of deleterious alleles via *cis*‐regulatory selection. Third, hybrids show reduced transcriptomic entropy and expanded gene regulatory networks, enhancing transcriptomic plasticity across developmental stages and environments. Collectively, these gene expression rebalancing, allele‐specific optimization, and transcriptomic plasticity drive heterosis and genetic gain in maize hybrid breeding.

Regulatory genetic variation has played a pivotal role in crop domestication and adaptation, with 64% of natural variants underlying cloned QTLs in maize attributable to regulatory changes (Chen et al. 2021). Comparative transcriptomic analyses have revealed restructuring of co‐expression networks during both maize domestication and improvement. Notably, the key regulatory genes responsible for these transcriptomic changes are functionally enriched in stress response pathways (Swanson‐Wagner et al. 2006; Liu et al. 2015). Furthermore, the variation in gene expression of hybrids and their parents has been characterised in different plants (Paschold et al. 2014; Klosinska et al. 2016; Waters et al. 2017). Although many traits showed superior performance in maize hybrids, the majority of gene expression patterns are additive in different tissues (Zhou et al. 2019) and different hybrids (Figure 2; Figure S6; Table S10). An interesting finding in the present study is that photosynthesis‐related genes showed bias toward overdominant expression while genes related to stress response were down‐regulated in hybrids compared to parents (Figure 2 and Figure S4). Our observation of expression trade‐off between photosynthesis‐related and stress‐responsive genes suggests a potential reprogramming of gene networks that enables hybrids to grow faster, accumulate more biomass, and yield more under different conditions (Figure 7). Consistently, previous studies have shown a correlation between enhanced photosynthesis and vegetative growth vigour in *Arabidopsis* hybrids (Fujimoto et al. 2012). Furthermore, the *Arabidopsis* hybrids exhibited high‐parent‐dominant expression complementation of hub regulatory genes within photosynthesis function (Liu et al. 2021). The stress‐related genes showed repressed expression under normal conditions but induced expression to mid‐parent or higher levels under stress in hybrids of *Arabidopsis* (Miller et al. 2015). Understanding the regulatory interplay between stress response and growth regulation pathways in maize will enable the design of stress‐resilient crop varieties that maintain high productivity under adverse environmental conditions.

Allele‐specific expression (ASE) represents an important regulatory mechanism in hybrids (Hochholdinger and Baldauf 2018). Guo et al. (2004) firstly reported the ASE patterns in plant hybrids, demonstrating the differential expression of parental alleles' responses to abiotic stresses in maize hybrids. Through a genome‐wide ASE analysis, we found that allele‐specific response to planting density stress was prevalent in maize hybrids (Figure 4; Figures S10B and S12). Large‐effect variants in the coding sequence of genes, such as protein‐truncating variants, frequently result in loss‐of‐function alleles and may show suppressed allelic expression (Rivas et al. 2015). Our results revealed that the proportion of deleterious allele suppression showed a significant positive correlation with hybrid yield increase during breeding improvement, supporting a major role for *cis*‐regulatory selection in hybrid breeding (Figures 5 and 7). Notably, complementation of deleterious variants in *cis*‐regulatory regions showed stronger selection signatures and yield correlations than coding variants (Figure S13), aligning with previous observations of selection signatures on *cis*‐regulatory regions during domestication and improvement of maize (Lemmon et al. 2014; Liu et al. 2015).

Since Darwin's initial documentation of heterosis (Darwin 1876), the genetic basis of this phenomenon has remained a subject of ongoing debate (Birchler 2015). The genetic basis of heterosis has been primarily explained through two classical models: the dominance hypothesis and overdominance hypothesis (Birchler 2015). While single‐locus overdominance can drive heterosis in specific cases, such as *SFT* in tomato (Krieger et al. 2010) and *CNR1* in maize (Guo et al. 2010), genome‐wide studies demonstrate that dominance is prevalent for loci associated with yield or other traits in large hybrid populations (Huang et al. 2015, 2016). However, these genetic models remain incomplete, prompting alternative frameworks like the gene balance hypothesis (Birchler et al. 2010) and protein quality control hypothesis (Goff and Zhang 2013) to address their limitations. Our study introduces entropy as a novel metric for heterosis, quantifying transcriptomic plasticity in hybrids across developmental stages and stress conditions. The significant correlation between entropy reduction and heterosis (Figure 6 and Figure S16) aligns with Schrödinger's concept of “negative entropy” as a biological organising principle (Schrödinger 1944). The lower transcriptomic entropy in hybrids, implying a departure from a uniform expression distribution toward a more structured one, likely reflects a more ‘orchestrated’ transcriptional program. This increased coordination may enhance the efficiency of resource allocation by reducing transcriptional noise and focusing expression on key pathways, thereby contributing to heterosis by providing greater phenotypic stability and adaptability. This transcriptional entropy reduction mechanism mirrors phenomena observed in other biological systems. By using entropy as a measure of transcriptional heterogeneity, Fennell et al. (2022) found that increased transcriptional heterogeneity enables the clonal fitness and competition of clinical mouse models with acute myeloid leukaemia, in diverse immune microenvironments. Cizeron et al. (2020) used entropy to estimate the synapse diversity in brain regions across the mouse life span, and found that expansion of synapse diversity created differentiation of brain regions until early adulthood and its reduction caused dedifferentiation in old age. The methylome entropy in the blood of naked mole‐rat showed a sharp decrease at youngest ages and increased during the whole lifespan (Kerepesi et al. 2022). These results suggested the diversity or heterogeneity at molecular level correlated with the vigour of living beings. Consistent with this notion, our results of the reduced entropy in the hybrid suggested that the dynamic transcriptomes generate more active or flexible gene regulatory networks and create superior fitness in hybrid (Figures 6 and 7; Figures S18–S20). Despite establishing a link between reduced transcriptomic entropy and heterosis, key questions remain for future research—particularly regarding the mechanisms underlying transcriptomic entropy reduction in hybrids and the potential role of key regulatory genes in this process.

## 4 Conclusion

In summary, this study provides a transcriptomic overview of the landmark hybrids and their parental inbreds in the history of hybrid maize development in China. We demonstrate the dynamics of gene and allele expression regulation throughout maize development and planting density adaptation. Our findings advance the molecular understanding of maize hybrid breeding improvement and heterosis, and establish a novel framework for molecular breeding that leverages expression optimization of key alleles to enhance hybrid performance.

## 5 Materials and Methods

### 5.1 Plant Materials and Field Experiment

The 20 hybrids analysed in this study represent landmark hybrids that have shaped the breeding history of single‐cross hybrids in China. The parental inbred constitutes elite germplasm that has been extensively utilised in Chinese maize breeding programs (Li et al. 2014; Table S1). A total of 33 traits were recorded under 37 500, 52 500 and 67 500 plants/ha at five environments for the hybrids and parental inbreds in our previous study (Li et al. 2014). The absolute heterosis for a given trait was calculated as the difference between the hybrid's phenotypic value and the mid‐parent value (the average value of the two corresponding parental lines). The percent heterosis was subsequently derived by dividing the absolute heterosis by the mid‐parent value. To estimate the genetic gain over time, a simple linear regression model was fitted with the trait performance or the heterosis value and the year of hybrid release.

Field experiments were conducted in the summer of 2020 at the Shunyi experimental station (116°34′ E, 40°13′N) of the Institute of Crop Sciences, Chinese Academy of Agricultural Sciences, Beijing. Two planting density treatments were established: 120 000 plants/ha (high planting density, HPD) and 33 000 plants/ha (low planting density, LPD). For the HPD treatment, the row and column spacing were set at 0.6 m and 0.15 m, respectively. In contrast, for the LPD treatment, both the row and column spacing were set at 0.6 m. Hybrids and parental lines were planted separately in four row plots with a row length 3.0 m. Three replicates were grown for each maize line and planting density treatment. To avoid border effects, the plot arrangements of different materials were sorted based on their plant height.

We selected three critical developmental stages representing distinct phases of maize growth: V4 (4‐leaf stage), V10 (10‐leaf stage), and VT (tasselling stage). The V4 represents the early vegetative phase with active leaf establishment. V10 marks the key reproductive development stage of tassel and ear. VT is the critical flowering stage determining pollination success. This selection allows comprehensive monitoring of transcriptome dynamics across vegetative growth, reproductive transition, and flowering phases. Leaf samples were collected at 21 (V4), 37 (V10), and 52 (VT) days after sowing. In the mornings at 9:00 to 11:00, the newest fully expanded leaves (visible of leaf sheath) from five randomly selected plants in the middle two rows of each plot were sampled. Leaf samples of 10 to 20 cm from the blade tip were collected and immediately stored in liquid nitrogen. Three biological replicates were collected for each genotype, planting density, and developmental stage, with each replicate consisting of leaf samples from five individual plants.

### 5.2 RNA Sequencing and Data Processing

The total RNA of 919 leaf samples was isolated using RNeasy Plant Mini Kit (Qiagen), with on‐column DNase digestion. The qualities of RNA samples were identified using Agilent 2100 (Agilent) and NanoDrop (Thermo Fisher). The mRNA sequencing libraries were prepared using Illumina Stranded mRNA Prep and sequenced on Illumina on NovaSeq 6000 System.

The 150‐bp paired‐end clean reads were generated after quality control using fastp v0.20.0 (Chen et al. 2018). We used HISAT2 v2.2.1 (Kim et al. 2019) to align the clean reads against the maize B73 reference genome (B73 RefGen_v4) (Jiao et al. 2017) with default parameters. The aligned reads were sorted and processed using SAMtools v1.11 (Li et al. 2009). The fragments per kilobase of exon model per million (FPKM) of each gene was calculated with StringTie v2.1.1 (Pertea et al. 2015).

In addition, the aligned RNA sequencing data were used for variant calling. We used MarkDuplicates from Picard v2.20.4 (http://broadinstitute.github.io/picard), SplitNCigarReads, BaseRecalibrator, ApplyBQSR, and HaplobypeCaller from GATK v4.1.2.0 (McKenna et al. 2010) for variant calling. The raw variants with FS > 60.0, QD < 2.0, MQ < 25.0, MQRankSum < −12.5 and ReadPosRankSum < −8.0 were retained to obtain high‐quality variants.

### 5.3 DNA Sequencing and Data Processing

For the parental inbred lines, we also performed DNA sequencing to identify genomic variants. Five young seedlings of each inbred were collected for sequencing. The genomic DNA was isolated using the cetyl trimethylammonium bromide (CTAB) method. DNA sequencing was performed on the NovaSeq 6000 System.

The paired‐end clean reads were obtained after quality control using FastQC v0.11.5 (https://www.bioinformatics.babraham.ac.uk/projects/fastqc/). The clean DNA sequencing data were aligned to B73 RefGen_v4 (Jiao et al. 2017) using BWA‐MEM v0.7.15 (Li and Durbin 2009) with default parameters. The aligned reads were sorted with SAMtools v1.1168 and the duplicate reads were marked using SAMBAMBA 0.6.6 (Tarasov et al. 2015). We used GATK v4.1.2.0 (McKenna et al. 2010) with the functions BaseRecalibrator, ApplyBQSR and HaplobypeCaller for variants calling. The high‐quality variants were obtained with the criteria FS > 60.0, QD < 2.0, MQ < 45.0, MQRankSum < −12.5 and ReadPosRankSum < −8.0 using VariantFiltration function in GATK. The variant files from RNA and DNA sequencing were merged with CombineGVCFs and GenotypeGVCFs functions in GATK using default parameters.

### 5.4 PCA and MDS

The FPKM values of all genes across 919 samples were used for principal components analysis (PCA) and multidimensional scaling (MDS) analysis. The PCA was performed using the prcomp function in R v3.6.3 (https://www.r‐project.org/). We used the edgeR v3.36.0 (https://git.bioconductor.org/packages/edgeR) package in R to conduct MDS analysis for all samples or each hybrid and the parental inbred lines.

### 5.5 Population‐Level Differentially Expressed Gene Analysis

We employed a multiple factor differentially expressed (DE) gene to characterise the population‐level gene expression changes along with different genotypes, planting density treatments or development stages. The formula is set as ~ V4/V10/VT + Hybrid/Inbred + HPD/LPD + Hybrid/Inbred:HPD/LPD to quantify the impact of different factors, including the interaction terms between genotype and plant density treatment, on gene expression. We used DESeq2 v1.50.2 (Love et al. 2014) to fit the model and test DE genes responsible for different factors. A gene was identified as DE genes according to the criteria of the false discovery rate (FDR) < 0.01 and log_2_(fold changes) > 1 or < −1.

### 5.6 Additive/Non‐Additive Expression Analysis

The DE genes between the female and male parents of each hybrid were identified using DESeq2 v1.50.2 (Love et al. 2014) with pairwise comparison. Genes with FDR < 0.01 were identified as differentially expressed. The parental DE genes were classified into two‐fold‐DE (DE_2), four‐fold‐DE (DE_4) and eight‐fold‐DE (DE_8) based on the log_2_(fold changes) of the comparisons between two parental lines. A special case of parental DE is defined as single parent expression (SPE) genes when FPKM of one parent < 0.1 and FPKM of the other plant > 1. The SPE genes with FPKM > 1 in the corresponding hybrid were identified as hybrid complementarily expressed (CE) genes.

The dominance effect/additive effect (D/A) values of gene expression in hybrid were calculated based on the formula:

$$D/A=\frac{{\text{FPKM}}_{F1}-\text{mean}\left({\text{FPKM}}_{P1},{\text{FPKM}}_{P2}\right)}{\max\left({\text{FPKM}}_{P1},{\text{FPKM}}_{P2}\right)-\text{mean}\left({\text{FPKM}}_{P1},{\text{FPKM}}_{P2}\right)}$$

where *P1* and *P2* are the parents of corresponding *F*_*1*_ hybrid. The method of gene expression inheritance in hybrid was referenced to Zhou et al. (2019). Briefly, the normalised read counts of each replicate of two parents and the corresponding hybrid were used to estimate the dispersion parameters by DESeq2 and fit to the additive, dominant or overdominant models using maximum likelihood estimation. The Bayesian information criterion (BIC) was used to assess the best fitted models and assign gene expression inheritance.

### 5.7 Gene Set Enrichment Analysis

Gene set enrichment analysis (GSEA) was performed using R packages AnnotationHub v3.4.0 (https://bioconductor.org/packages/release/bioc/html/AnnotationHub.html) and clusterProfiler v4.2 (Xu et al. 2024). We selected org.zea_mays.eg.sqlite database as gene annotation references. The gene ID was transformed based on the tool of Translate Gene Model IDs in MaizeGDB (https://maizegdb.org). The GO‐based GSEA was performed based on the log_2_(fold changes) of DE analysis or the log_2_(D/A) ratio of genes in each hybrid using gseGO function in clusterProfiler package with “ont=All pAdjustMethod=BH pvalueCutoff = 0.05”.

### 5.8 Population Level Allele‐Specific Expression Analysis Based on Heterozygous Variants

A Bayesian generalised linear mixed model (McCoy et al. 2017) was employed for population level allele‐specific expression analysis. Only SNP variants were utilised for allele‐specific expression analysis to avoid the potential mapping bias caused by indels (Castel et al. 2016; Zhou et al. 2019). First, the allele‐specific read count of each genic SNP in hybrids was obtained using ASEReadCounter of GATK v4.1.2.0 (McKenna et al. 2010). The SNPs that were heterozygous in at least 10 hybrids and had at least 10 total read counts by ASEReadCounter were used for further analysis. The ASE read count of each SNP was used to fit with a binomial mixed model

$$\text{Logit}\left(p_{\mathrm{gt}}\right)=\beta_{0}+\alpha_{g}+\gamma_{t}$$

where *p*_*gt*_ is the proportion of ASE read count supporting the alternative variant in genotype *g* and planting density treatment *t*, and *α*_*g*_ and *γ*_*t*_ are random effects of genotype and treatment, respectively. The bias of ASE is estimated by the posterior probability distribution of the intercept term *β*_*0*_. The median ASE value of all observations in the filtered dataset is set as null hypothesis (0.4675). The posterior predictive *p*‐values were calculated based on the null hypothesis and the Benjamini‐Hochberg procedure was used to control the FDR at the 0.05 level.

### 5.9 Allele‐Specific Expression Analysis Based on Haplotypes for Each Hybrid

For each hybrid, the SNP genotype was manually phased based on their parents as follow. First, we removed the variants that were heterozygous in either parental line. And the conflicting variants between hybrid and parental lines were also removed. For instance, a variant that both parental lines are homozygous 0/0 genotype while the hybrid is heterozygous 0/1 genotype would be removed. Next, we manually phased the heterozygous variants in the hybrid based on their parental lines. Specifically, a variant was defined as 0|1 if the female parent is 0/0 and the male parent is 1/1, and as 1|0 when the genotypes of the parents were opposite.

The manually phased genotypes and aligned reads from RNA sequencing were used to conduct a subsequently allele‐specific expression analysis. We employed phASER v0.9.9.4 (Castel et al. 2016) to estimate the haplotype allele‐specific expression. This method reduced the false positive results of allele‐specific expression by addressing the cumulative counting issue that arises when a read overlaps multiple heterozygous variants. For all samples, parameters for phASER were set as follows: alignment score quantile cutoff of 0.05, MAPQ of 60, BASEQ of 10, and conflicting configuration threshold of 0.01. Additionally, the method for statistical identification of *cis*‐ or *trans*‐regulatory patterns was according to Zhou et al. (2019).

To investigate the allele‐specific expression response to density stress in hybrids, we used haplotype level allele expression values that obtained from phASER as input data and employed DESeq2 v1.50.2 (Love et al. 2014) to analyse allelic differential expression by comparing samples grown under different plant densities. Alleles exhibiting log_2_(fold change) > 1 or < −1 and FDR < 0.01 were identified as responsive to density stress.

### 5.10 Identification of Deleterious Alleles

The genetic variants of all maize lines obtained by RNA and DNA sequencing were annotated using SnpEff v5.0e (http://pcingola.github.io/SnpEff/). We selected the genes with high effect mutations (including SNPs and indels with frameshift, stop gained, splice acceptor, splice donor, gene fusion, exon loss, start lost, stop lost, or transcript ablation), which usually had large effects on protein function and phenotypic performance, for further analysis. For each hybrid, heterozygous genes in which one allele has one or more deleterious variants in coding regions and the other allele maintains a functional coding sequence were used for allele‐specific expression analysis. Genes with their expression of deleterious allele repressed [log_2_(allelic expression ratio) > 1 or < −1, and Binomial test FDR < 0.05] were classified as deleterious allele suppression.

In addition, the deleterious variants were also analysed using genomic evolutionary rate profiling (GERP) of maize (Swarts et al. 2017; Yang et al. 2017). Only the SNPs with rejected substitution (RS) scores > 2 were filtered for subsequent analysis. The complementation of deleterious variants in hybrid were estimated according to the genotypes of two parental inbred lines. And the complemented deleterious variants were separated into coding regions and *cis*‐regulatory regions (2‐kb upstream of gene), respectively.

### 5.11 eGWAS Analysis

We performed an eGWAS analysis on expression data from 52 maize lines across three development stages and two planting density treatments using eQtlBma v1.3.3 (Flutre et al. 2013). The mean expression value of each gene was calculated from replicate samples. Genes with an average expression level (FPKM) < 1 across development stages and treatments were filtered out as lowly expressed. The genomic SNPs with missing rate > 20% and MAF < 0.05 were removed for further analysis. Expression data were normalised using rank‐based inverse normal transformation (INT) to eliminate the effects of scale differences. The scanning window was set to 1 Mb upstream and downstream of each gene, and eGWAS analysis was performed using linear regression model, with a significant eQTN threshold set at log_10_(Bayes Factors, BF) > 5. The configuration competition model was used to identify conserved eQTN signals across different developmental stages and treatments. Subsequently, *cis*‐QTNs located in the promoter region (2‐kb region upstream of each gene) were selected for comparative analysis with genomic regions under breeding selection (Li et al. 2022). Statistical enrichment was tested using Fisher's Exact Test.

### 5.12 Transcriptomic Entropy Analysis

To estimate the global diversity of transcriptomes during development and stress response, we introduced a new concept and defined the transcriptomic dynamic as the Shannon's entropy of its frequency distribution in each genotype. First, the transcriptomes of each genotype under different plant densities and developmental stages were filtered using goodSamplesGenes and cutreeStatic functions in R package WGCNA v1.73 (Langfelder and Horvath 2008). Then, the expression frequency of a single gene was calculated as

$$p_{i}=\frac{1}{t}\sum_{j=1}^{t}p_{\mathrm{ij}}$$

where the average frequency of the gene *i* equals the mean of expression frequencies in samples (*j* = 1, 2, …, t) of a hybrid or parental inbred line. Then the transcriptomic dynamics (Shannon's entropy) of line *G* was estimated as

$$H_{G}=-\sum_{i=1}^{g}p_{i}\log_{2}\left(p_{i}\right)$$

where the *i* presents expressed gene (total number of *g*). The entropy reduction was calculated based on the difference between hybrids and their parental inbred lines.

### 5.13 Identification of Co‐Expression Clusters

We used hierarchical clustering analysis to identify the co‐expression clusters in each genotype. The FPKM of filtered transcriptomes was used to build Pearson's correlation coefficient‐based distance matrix. The hierarchical clustering was performed using hclust function in R with “method = ward.D2”. The clustering result was cut using cutreeDynamic function in R package dynamicTreeCut v1.63 (Langfelder et al. 2008) with deepSplit of 1 and similar clusters were merged with cutHeight of 0.15, 0.2 and 0.25, respectively.

### 5.14 Gene Regulatory Network Construction

For each genotype, the filtered transcriptomes in different developmental stages and planting density treatments were used for gene regulatory network (GRN) construction. The transcription factor genes were downloaded from PlantTFDB database (Jin et al. 2017). GRNs were constructed using GRNBoost2 algorithm in Arboreto framework (Moerman et al. 2019). GRNBoost2 is based on Stochastic Gradient Boosting Machine regression and developed a self‐tuning mechanism for estimation of decision trees number in boosting ensembles. The seed value for initialization of machine learning algorithms was set to 42 for all GRNs. And all the GRNs were cut off by the link importance of 10 for comparisons.

## Author Contributions

T.W., M.G., Y.L. and X.L. conceived and designed the experiments. X.L., Yongxiang L., C.L., D.Z., G.H., H.L. and S.S. performed the experiment. X.L. performed bioinformatics analyses of data. Yongxiang L. and C.L. participated in data analysis. X.L. wrote the original manuscript. T.W., M.G. and Y.L. revised the manuscript. All authors have read and approved the final manuscript.

## Funding

This work was supported by National Key Research and Development Program of China, 2021YFD1200700. China Agriculture Research System, CARS‐02‐04, CARS‐02‐03. Innovation Program of Chinese Academy of Agricultural Sciences, CAAS‐CSCB‐202403.

## Conflicts of Interest

The authors declare no conflicts of interest.

## Supporting information


**Figure S1:** Heterosis levels and gene expression profiles of maize hybrids.
**Figure S2:** PCA of transcriptome profiles of each hybrid and its corresponding parents.
**Figure S3:** MDS of transcriptome profiles of each hybrid and its corresponding parents.
**Figure S4:** DE of photosynthesis and stress response related genes between hybrids and parents.
**Figure S5:** Correlation analysis of genetic distance and transcriptomic differences in hybrids and their parents.
**Figure S6:** The additive and non‐additive expression patterns in hybrids.
**Figure S7:** ASE of photosynthesis and stress response related genes.
**Figure S8:** SNP‐based and haplotype‐based ASE in maize hybrids.
**Figure S9:** Distribution of allelic expression ratio in maize hybrids.
**Figure S10:** Distribution of allele‐specific expression patterns in maize hybrids.
**Figure S11:** ASE patterns in each maize hybrid.
**Figure S12:** Allelic expression response to planting density stress.
**Figure S13:** The complemented deleterious sites at *cis*‐regulatory regions and CDS regions during maize hybrid improvement.
**Figure S14:** Comparison of *cis*‐eQTN and breeding selection signatures in maize.
**Figure S15:** Transcriptomic dynamics in maize hybrids and parental inbreds.
**Figure S16:** Correlation between transcriptomic entropy reduction and phenotypic variation in hybrids.
**Figure S17:** Transcriptomic entropy of *Arabidopsis* ecotype Col‐0, Per‐1 and their F_1_ hybrid.
**Figure S18:** Expanded gene regulatory networks in maize hybrids.
**Figure S19:** Wired gene number in GRNs of maize hybrids and parental inbreds.
**Figure S20:** Wires in GRNs of maize hybrids and parental inbreds.
**Figure S21:** Similarity and diversity of wired genes in GRNs of maize hybrids and parental inbreds.
**Figure S22:** Similarity and diversity of wires in GRNs of maize hybrids and parental inbreds.

**Table S1:** List of maize hybrids and their parental inbreds.
**Table S2:** Sequencing data statistic.
**Table S3:** Statistic of sequencing reads alignment.
**Table S4:** DE gene between high and low planting densities in hybrids.
**Table S5:** DE gene between high and low planting densities in parental lines.
**Table S6:** DE gene between hybrids and parental lines.
**Table S7:** GSEA results of DE genes between hybrids and parental lines.
**Table S8:** GSEA results of DE genes between high and low planting densities in hybrids.
**Table S9:** GSEA results of DE genes between high and low planting densities in parental lines.
**Table S10:** Statistic of parental DE genes assigned to additive or non‐additive expression patterns in hybrids.
**Table S11:** Significant ASE SNPs and genes detected by Bayesian GLMM in hybrids.
**Table S12:** Overlap of genes with significant ASE SNPs and convergent or co‐directional selected genes in female and male heterotic groups.
**Table S13:** Analysis of allelic expression patterns in hybrids.
**Table S14:** Analysis of ASE directions in adaption to high and low planting densities in hybrids.
**Table S15:** Summary of allelic response to planting densities in hybrids.

## Data Availability

The raw RNA and DNA sequencing data were deposited in the Genome Sequence Archive (GSA, https://bigd.big.ac.cn/gsa) under the Accession Code CRA025171 and CRA026071, respectively. The DNA sequencing data of 450 maize inbred lines are downloaded from PRJCA009749 of GSA. The codes used in this study are available at https://github.com/leo‐caas/ase_maize_hybrid.
