# Supplementary material for: Dynamics of Gene and Allelic Expression During Modern Hybrid Maize Breeding
Source: Plant Biotechnol J. 2026 Feb 26;24(6):3922–38. doi: 10.1111/pbi.70602 (PMC13205921; doi:10.1111/pbi.70602)
Supplement: Supplementary file 1 — Figure S1: Heterosis levels and gene expression profiles of maize hybrids. Figure S2: PCA of transcriptome profiles of each hybrid and its corresponding parents. Figure S3: MDS of transcriptome profiles of each hybrid and its corresponding parents. Figure S4: DE of photosynthesis and stress response related genes between hybrids and parents. Figure S5: Correlation analysis of genetic distance and transcriptomic differences in hybrids and their parents. Figure S6: The additive and non‐additive expression patterns in hybrids. Figure S7: ASE of photosynthesis and stress response related genes. Figure S8: SNP‐based and haplotype‐based ASE in maize hybrids. Figure S9: Distribution of allelic expression ratio in maize hybrids. Figure S10: Distribution of allele‐specific expression patterns in maize hybrids. Figure S11: ASE patterns in each maize hybrid. Figure S12: Allelic expression response to planting density stress. Figure S13: The complemented deleterious sites at cis‐regulatory regions and CDS regions during maize hybrid improvement. Figure S14: Comparison of cis‐eQTN and breeding selection signatures in maize. Figure S15: Transcriptomic dynamics in maize hybrids and parental inbreds. Figure S16: Correlation between transcriptomic entropy reduction and phenotypic variation in hybrids. Figure S17: Transcriptomic entropy of Arabidopsis ecotype Col‐0, Per‐1 and their F1 hybrid. Figure S18: Expanded gene regulatory networks in maize hybrids. Figure S19: Wired gene number in GRNs of maize hybrids and parental inbreds. Figure S20: Wires in GRNs of maize hybrids and parental inbreds. Figure S21: Similarity and diversity of wired genes in GRNs of maize hybrids and parental inbreds. Figure S22: Similarity and diversity of wires in GRNs of maize hybrids and parental inbreds. [file PBI-24-3922-s001.docx]

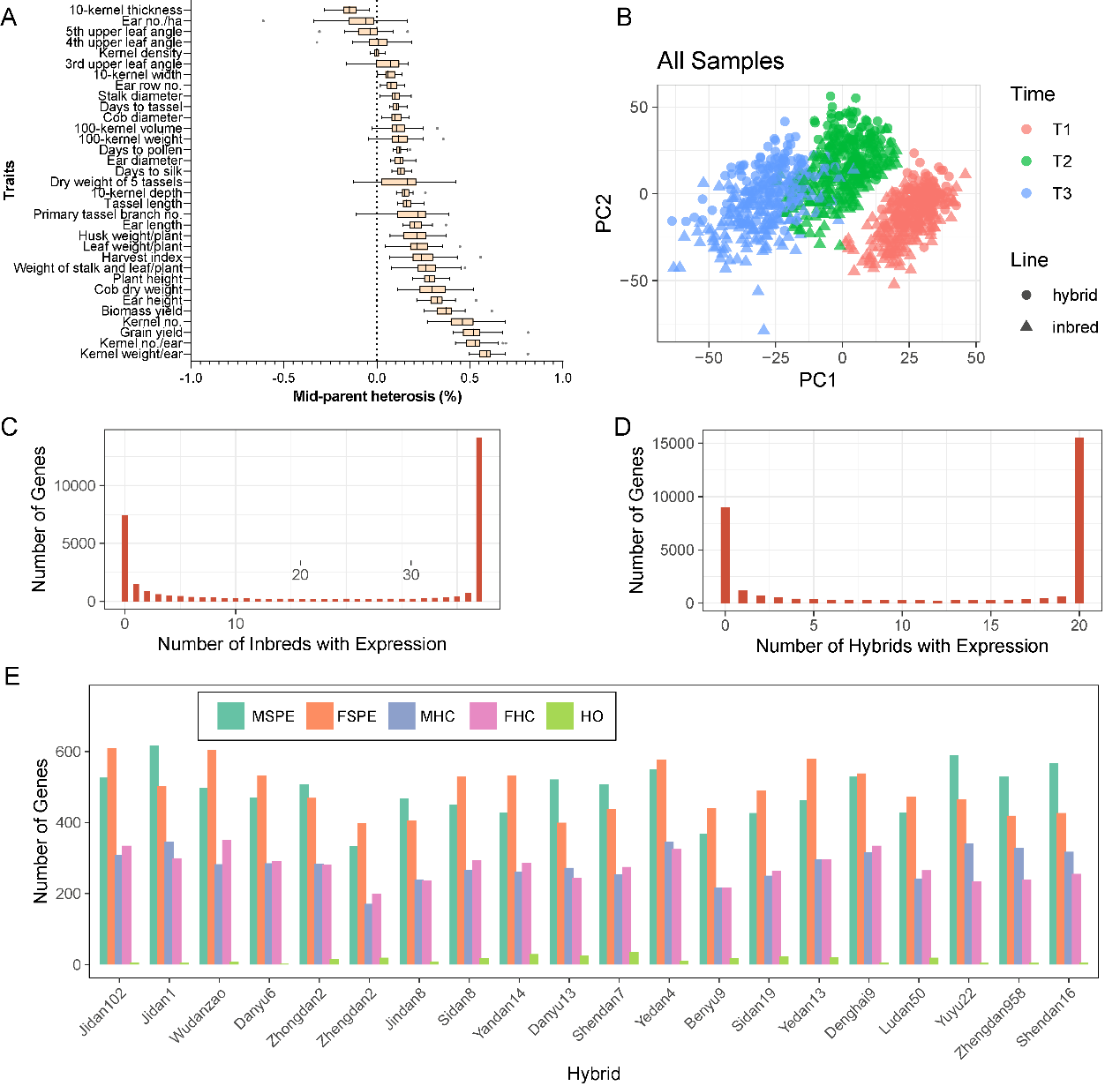


**Figure S1.** **Heterosis levels and gene expression profiles of maize hybrids. (A)** The percent heterosis of 33 traits in 20 maize hybrids. The data have been published in our previous studies (Li et al., 2014). **(B)** Principal component analysis (PCA) of the transcriptome profiles of maize hybrids and parental inbred lines. Different colors represent development stages V4 (T1), V10 (T2) and VT (T3). Dots and triangles are maize hybrids and parental inbred lines, respectively. **(C)** Number of expressed genes among 32 inbred lines. **(D)** Number of expressed genes among 20 hybrids. **(E)** Distribution of SPE and CE genes in 20 hybrids and corresponding parents. MSPE and FSPE mean male and female single parent expression, respectively. MHC and FHC mean male and female SPE genes that showed hybrid complementation, respectively. HO, hybrid expression only.


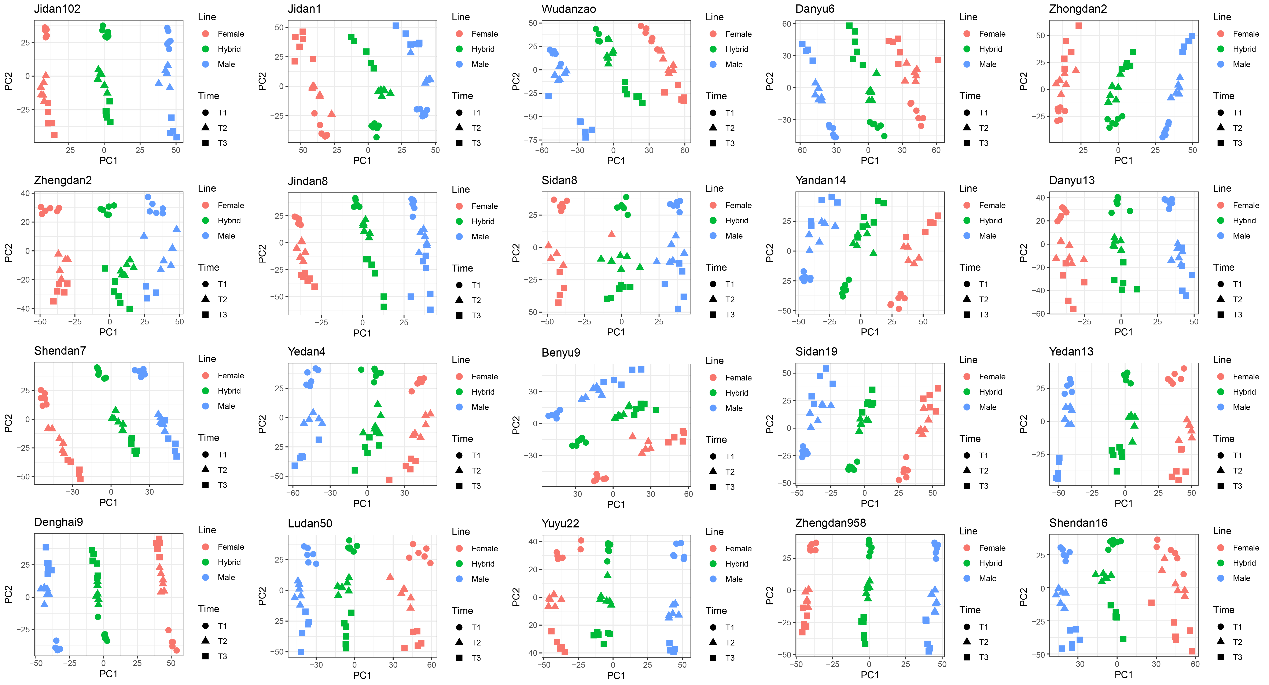


**Figure S2. PCA of transcriptome profiles of each hybrid and its corresponding parents.** Different colors indicate the hybrids and parental inbred lines. Different shapes represent of development stages V4 (T1), V10 (T2) and VT (T3).


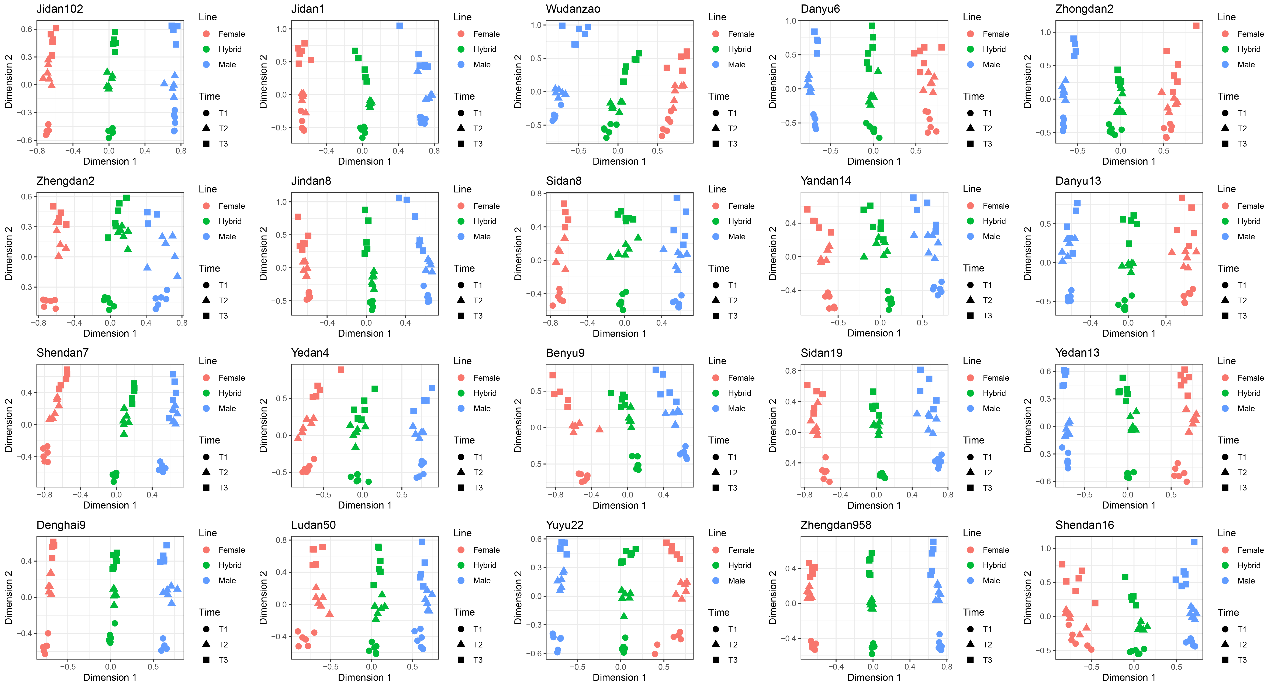


**Figure S3. MDS of transcriptome profiles of each hybrid and its corresponding parents.** Different colors indicate the hybrids and parental inbred lines. Different shapes represent development stages V4 (T1), V10 (T2) and VT (T3).


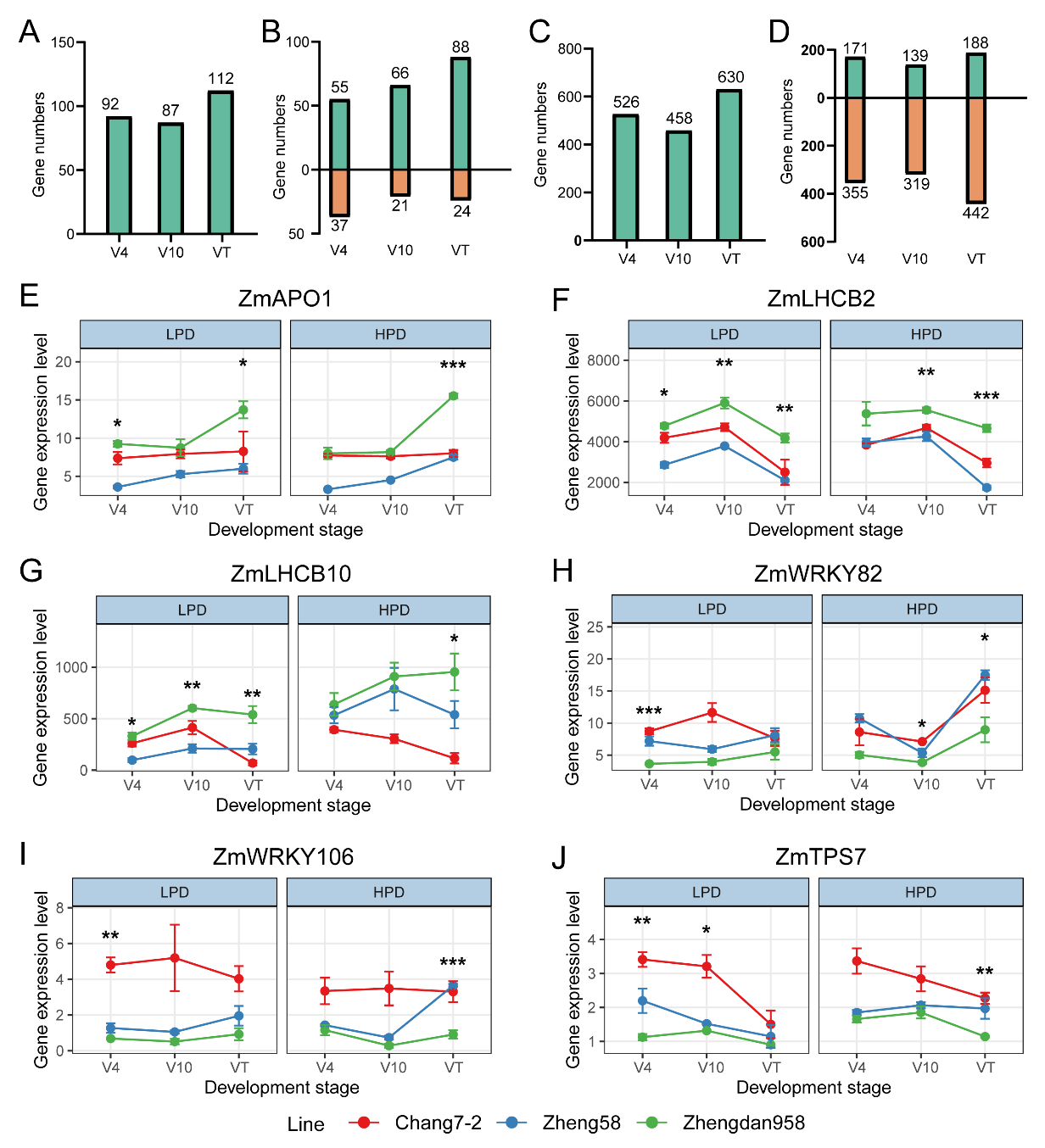


**Figure S4. DE of photosynthesis and stress response related genes between hybrids and parents.** **(A)** Number of differentially expressed (DE) genes of stress response category between hybrids and inbreds with multiple-factor DE gene analysis. **(B)** Number of up- (green) or down-regulated (orange) DE genes of stress response category between hybrids and inbreds. **(C)** Number of DE genes of photosynthesis related category between hybrids and inbreds. **(D)** Number of up- (green) or down-regulated (orange) DE genes of photosynthesis related category between hybrids and inbreds. **(E-F)** Expression profile of *ZmAPO1*, *ZmLHCB2*, *ZmLHCB10*, *ZmWTRY82*, *ZmWRKY106* and *ZmTPS7* in hybrid Zhengdan958 and parental inbreds Zheng58 and Chang7-2. LPD and HPD present low and high planting densities, respectively. The difference significance was estimated between hybrid and parents with Student’s *t*-test. *: *P* < 0.05, **: *P* < 0.01, ***: *P* < 0.001.


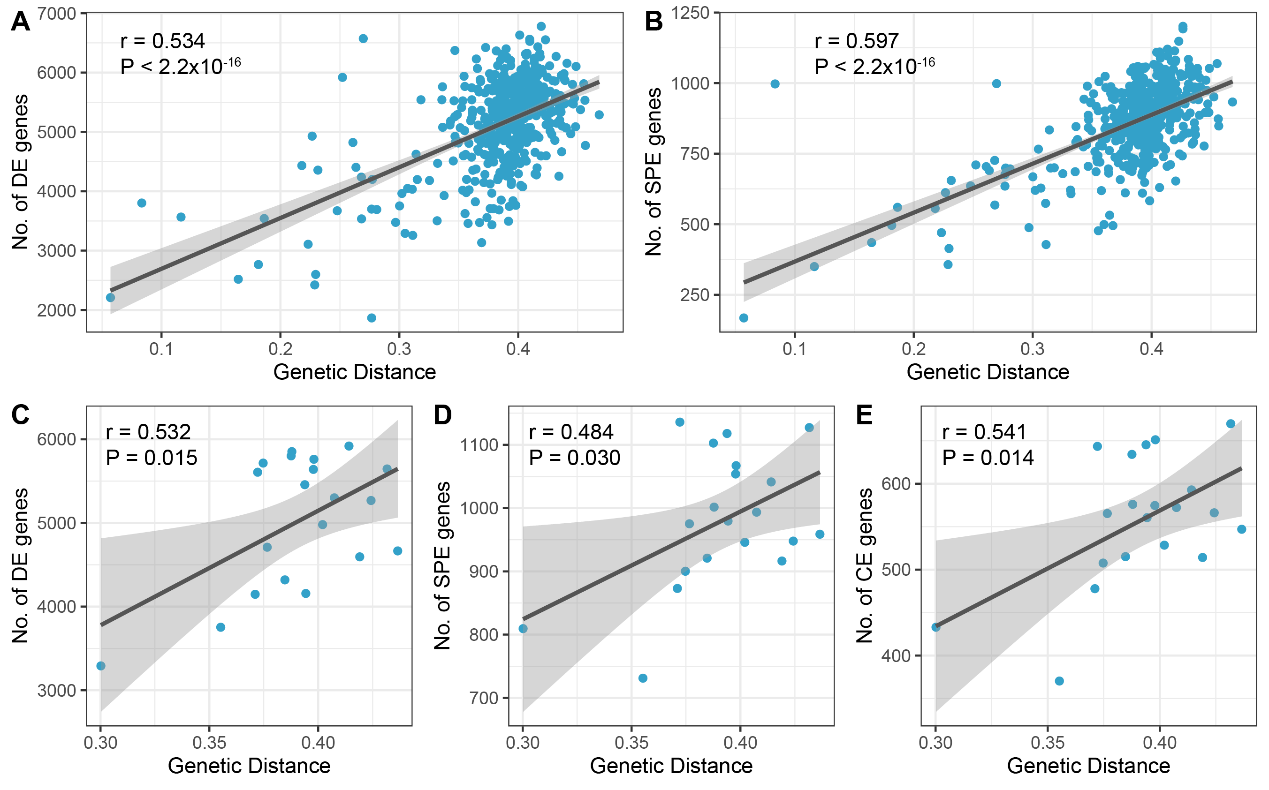


**Figure S5. Correlation analysis of genetic distance and transcriptomic differences in hybrids and their parents. (A)** Correlation of genetic distance and number of DE genes in all inbred line pairs. **(B)** Correlation of genetic distance and number of single-parent expression genes in all inbred line pairs. **(C)** Correlation of genetic distance and number of DE genes in the corresponding parent pairs of 20 hybrids. **(D)** Correlation of genetic distance and number of single-parent expression genes in the corresponding parent pairs of 20 hybrids. **(E)** Correlation of genetic distance between parents and the number of complementally expressed genes in hybrids.


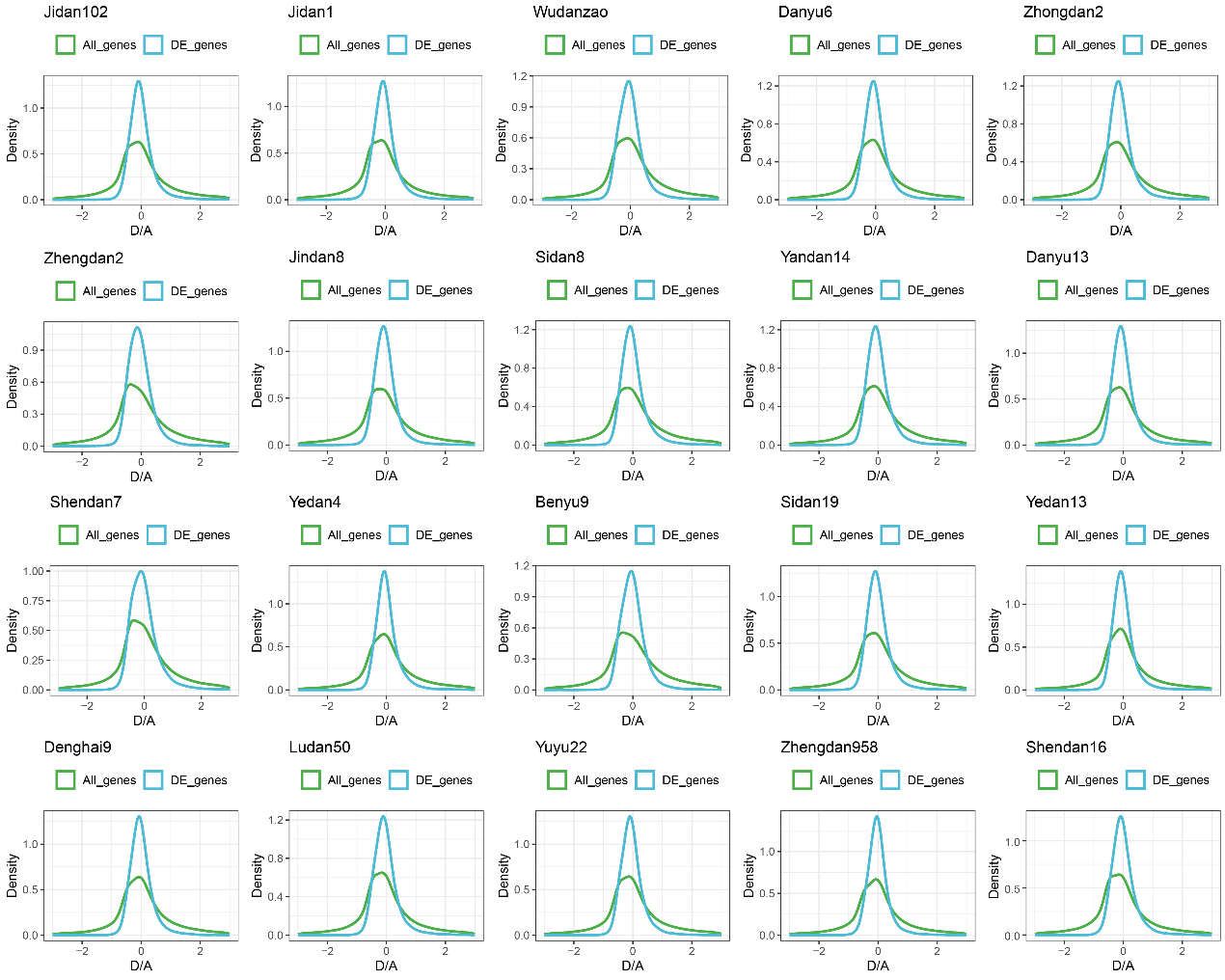


**Figure S6. The additive and non-additive expression patterns in hybrids.** Density distribution of dominance/additivity (D/A) ratios of all expressed genes and parental DE genes (labeled with different line color) across hybrids and corresponding parents.


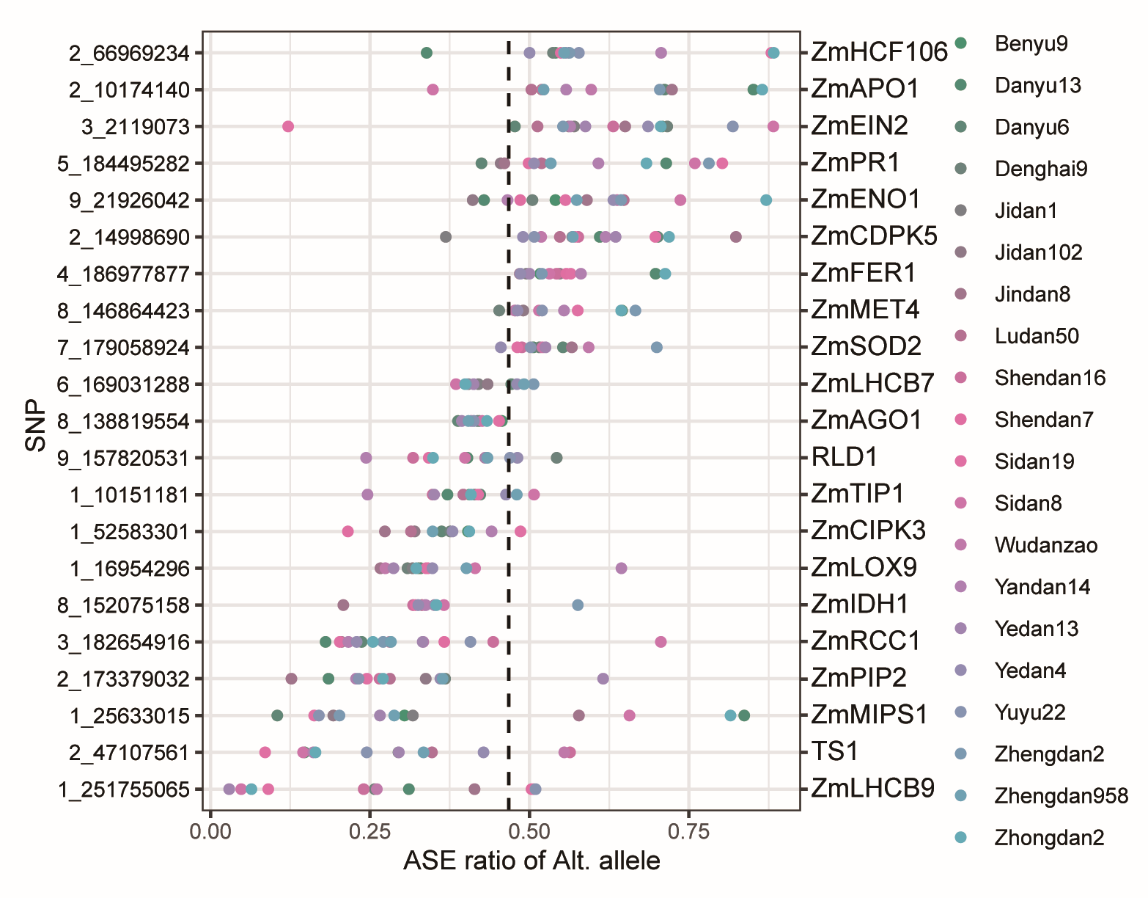


**Figure S7. ASE of photosynthesis and stress response related genes.** Twenty-one selected genes with significant ASE SNPs are shown with ASE ratio of different hybrids. The tags in left panel of y axis indicate ASE SNPs, which are in format of chromosome and position. The tags in right panel of y axis indicate gene within significant ASE SNPs.


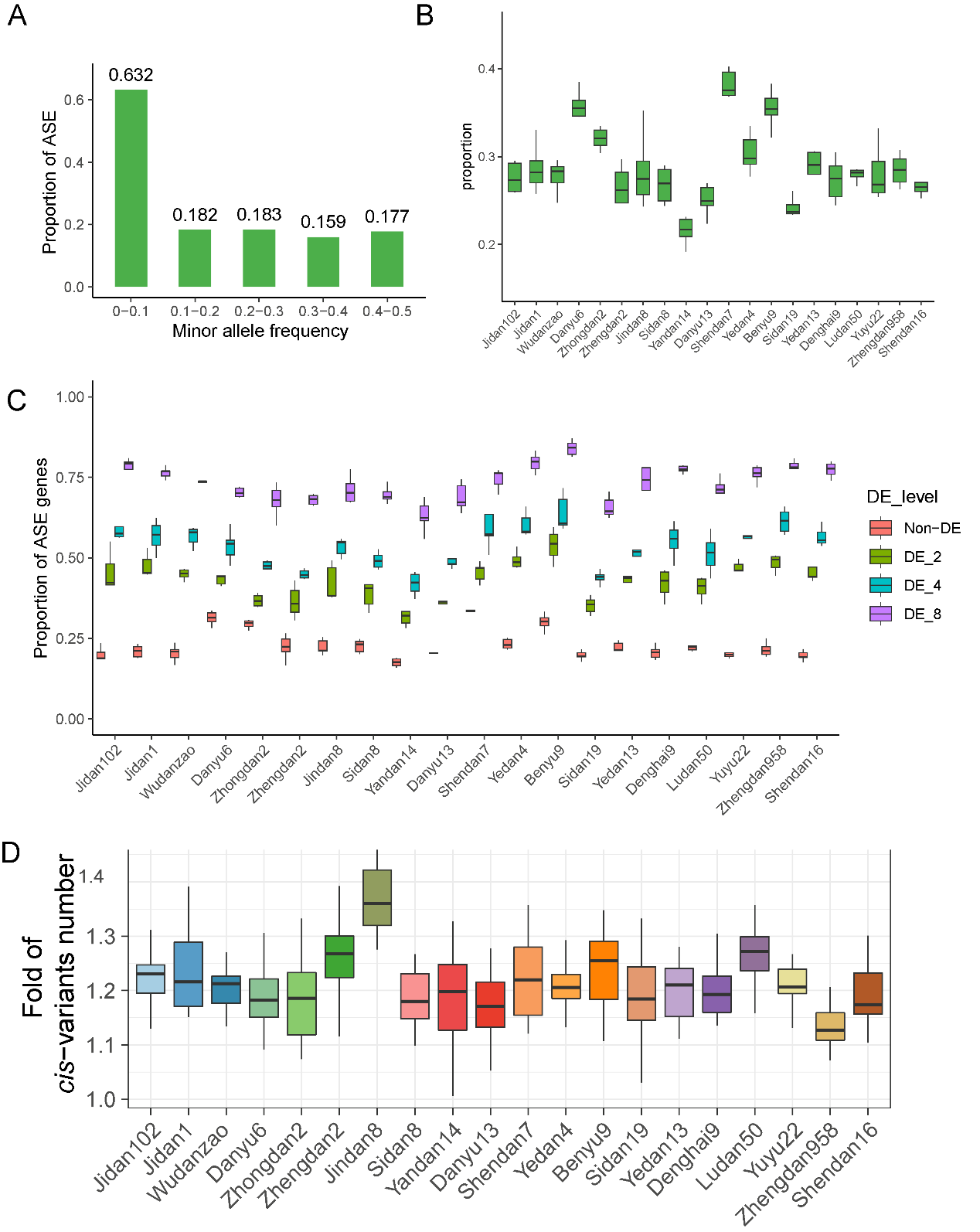


**Figure S8. SNP-based and haplotype-based ASE in maize hybrids. (A)** Proportion of variants showed significant ASE along with different minor allele frequency (MAF). The variants are different categories based on their MAF obtained from 1606 maize inbred lines. The proportion of ASE within different MAF intervals is displayed. **(B)** Haplotype-based ASE in each hybrid. The expression of two alleles in hybrid was estimated by haplotype analysis and significant ASE was determined using binomial test. **(C)** Haplotype based ASE of parental DE genes in each hybrid. The average proportion of hybrid significant ASE genes with different DE level in parents. The DE_2, DE_4, and DE_8 include genes that showed significantly DE with absolute value of log2(fold change) in range of 1 to 2, 2 to 3 and above 3, respectively. **(D)** Comparison of genetic variants in *cis*-regulatory regions between imbalanced and balanced expression genes. The number of genetic variants in *cis*-regulatory regions, which included the upstream 2 kb region of transcription start site of each gene, were compared between genes for imbalanced and balanced expression. Fold changes in *cis*-variants number of the genes with imbalanced to balanced expression in 20 maize hybrids were shown using boxplot.


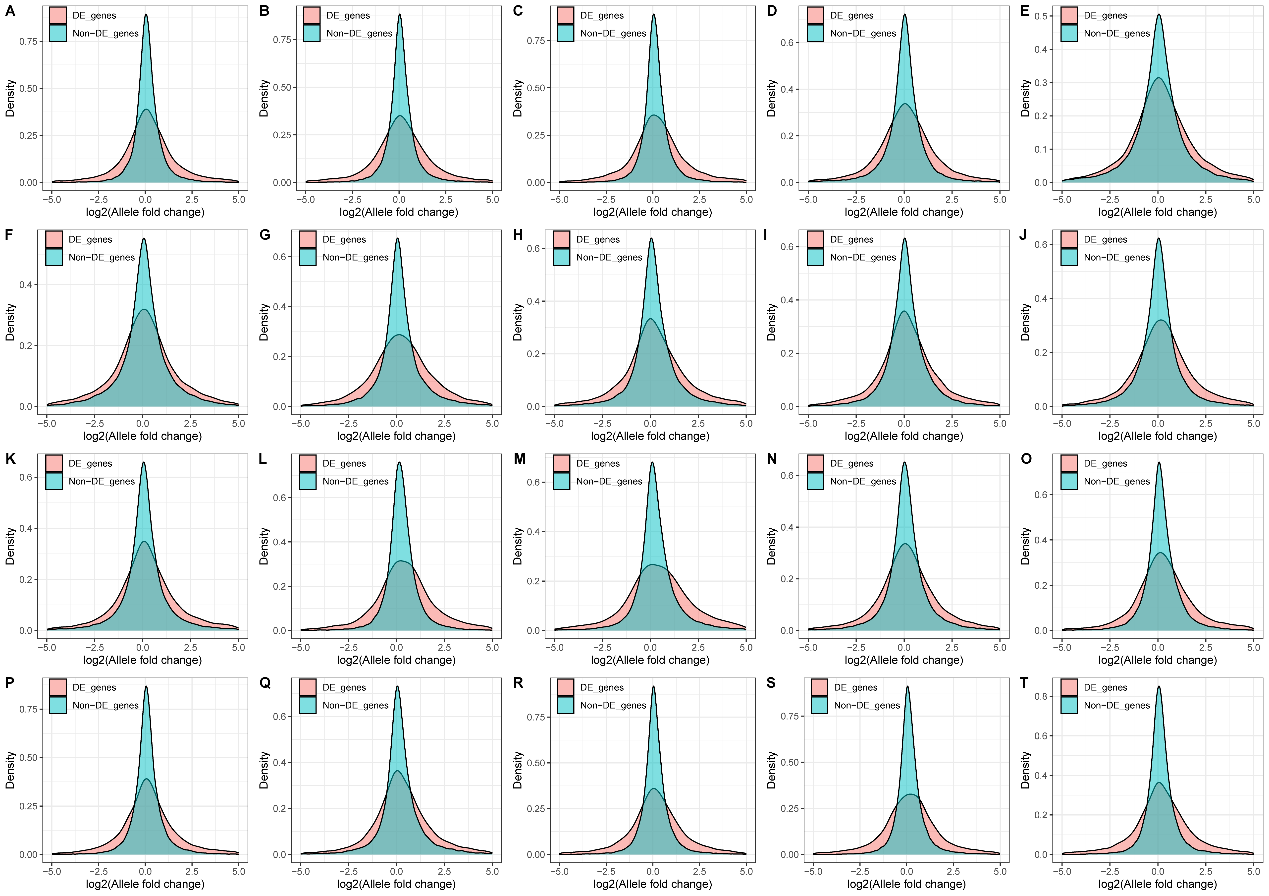


**Figure S9. Distribution of allelic expression ratio in maize hybrids. (A-T)** Distribution of allelic expression ratios in Jidan102, Jidan1, Wudanzao, Danyu6, Zhongdan2, Zhengdan2, Jindan8, Sidan8, Yandan14, Danyu13, Shendan7, Yedan4, Benyu9, Sidan19, Yedan13, Denghai9, Ludan50, Yuyu22, Zhengdan958, and Shendan16, respectively. Density distributions of allelic expression ratios of parental DE (orange) and non-DE (green) genes were shown.


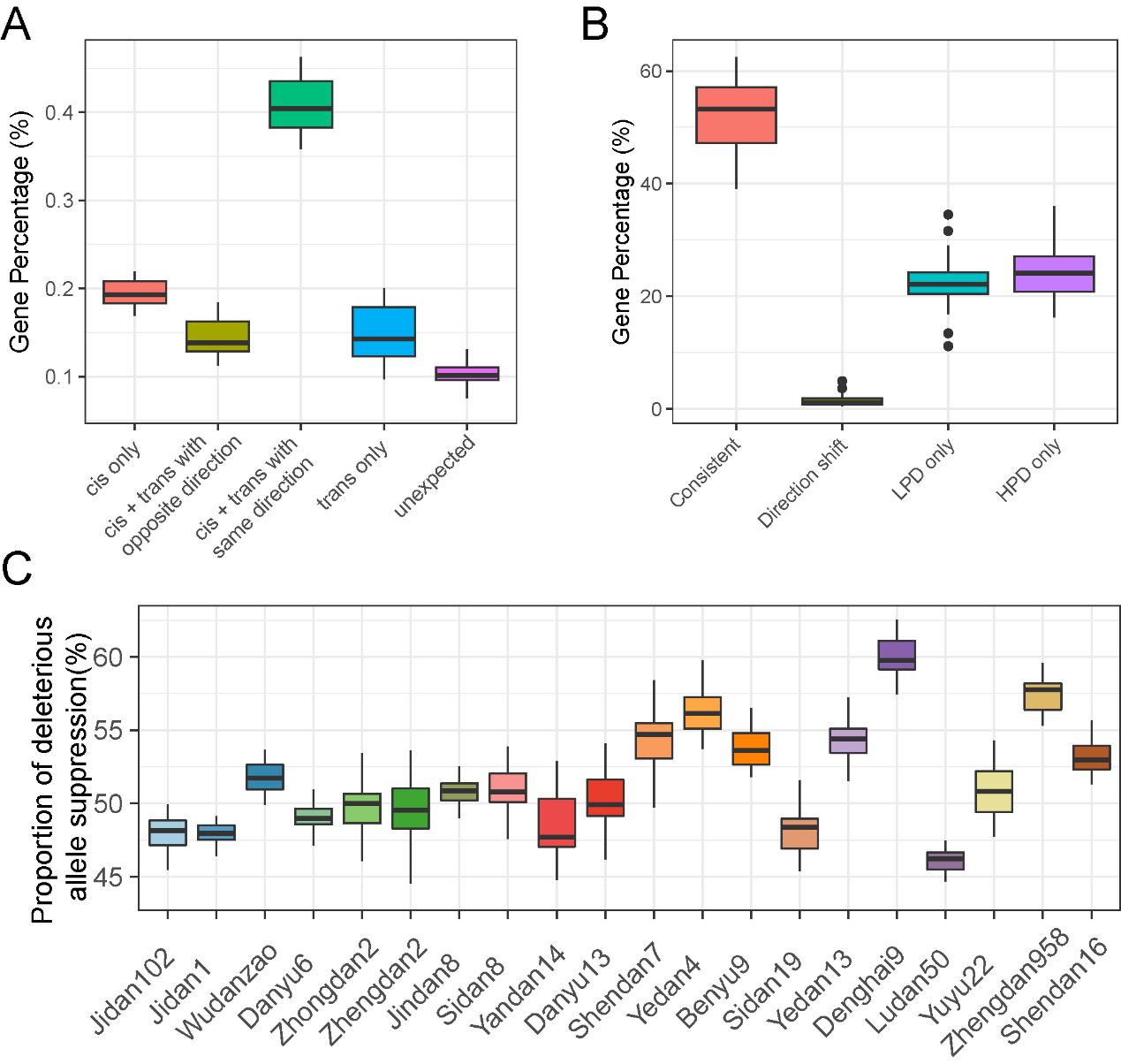


**Figure S10. Distribution of ASE patterns in maize hybrids. (A)** Percentage of genes showed different ASE patterns in hybrids. ASE genes in hybrids were estimated with *cis*- and *trans*-regulation effect. The genes showed same allelic expression ratio with parental DE ratio were classified as *cis*-only pattern. Genes that showed equal allelic expression were assigned to *trans*-only pattern. The *cis* + *trans* with same direction pattern included genes that showed lower ASE ratio in hybrid than parental DE fold change, while the genes with high ASE ratio in hybrid than parental DE fold change were classified as *cis* + *trans* with opposite direction pattern. The unexpected pattern indicates allelic expression ratio of a gene is the exact opposite of parental DE fold change. **(B)** Percentage of genes showed shifting ASE to planting density stress in hybrids. The consistent genes showed same allelic expression bias under HPD and LPD. The LPD only and HPD only genes showed only significant ASE under LPD or HPD, respectively. The direction-shifting genes showed higher expression of one parental allele under LPD while biased toward the other parental allele under HPD. **(C)** Proportion of deleterious allele suppression in maize hybrids. Deleterious allele suppression was defined as the expression level of predicted deleterious allele was significantly (Binomial test FDR < 0.05) lower than the functional allele in hybrid. The boxplot showed the distribution of deleterious allele suppression across different development stages and planting densities.


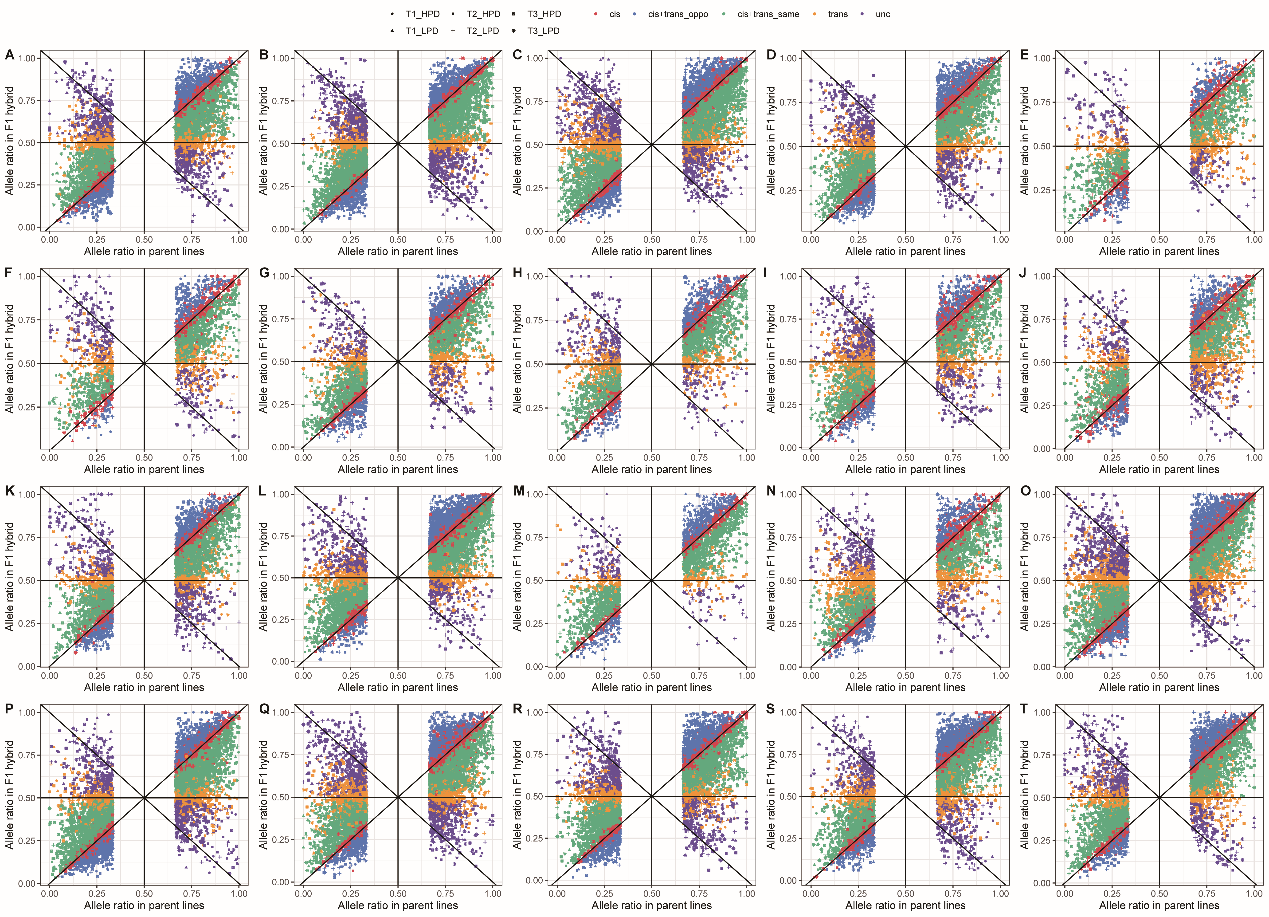


**Figure S11. ASE patterns in each maize hybrid. (A-T)** Allelic expression patterns in Jidan102, Jidan1, Wudanzao, Danyu6, Zhongdan2, Zhengdan2, Jindan8, Sidan8, Yandan14, Danyu13, Shendan7, Yedan4, Benyu9, Sidan19, Yedan13, Denghai9, Ludan50, Yuyu22, Zhengdan958, and Shendan16, respectively. The y axis presents allelic expression ratio of alleles from female and male parents. The x axis indicates the parental DE ratio between female and male parents.


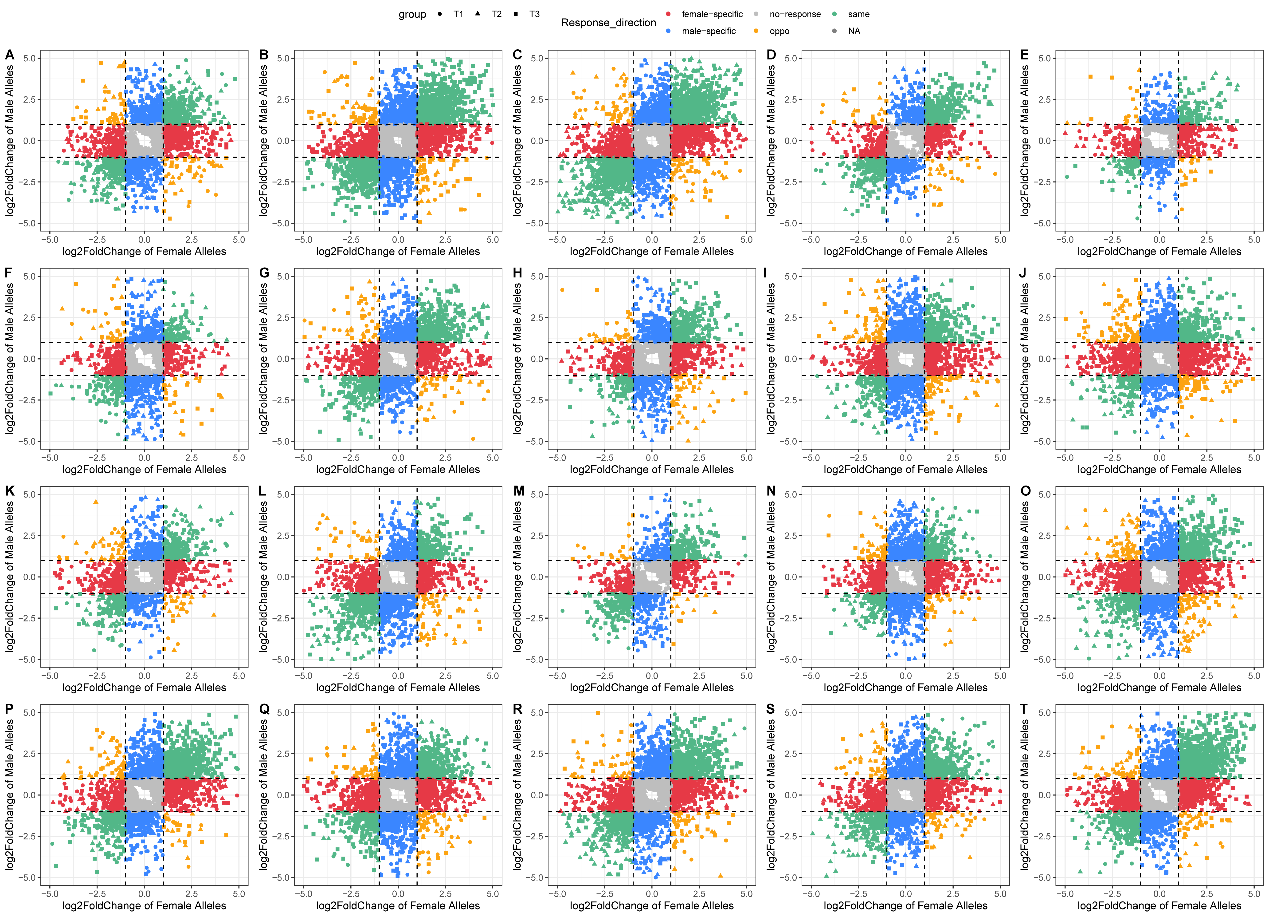


**Figure S12. Allelic expression response to planting density stress. (A-T)** Allelic response patterns in Jidan102, Jidan1, Wudanzao, Danyu6, Zhongdan2, Zhengdan2, Jindan8, Sidan8, Yandan14, Danyu13, Shendan7, Yedan4, Benyu9, Sidan19, Yedan13, Denghai9, Ludan50, Yuyu22, Zhengdan958, and Shendan16, respectively. The expression response of the two alleles to planting density stress was estimated separately. Based on the response direction of the two alleles, genes were assigned into different allelic response patterns, which is highlighted in different colors. Different shapes represent development stages V4 (T1), V10 (T2) and VT (T3).


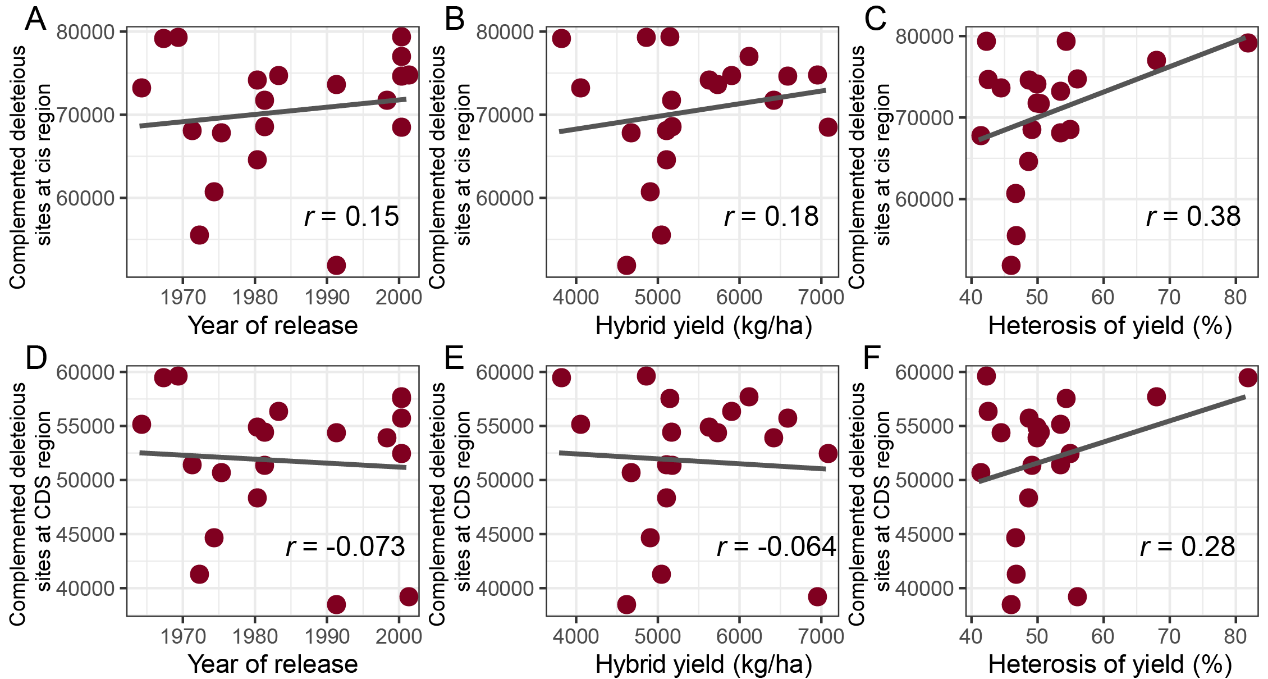


**Figure S13.** **The complemented deleterious sites at *cis*-regulatory regions and CDS regions during maize hybrid improvement. (A)** Historical changes of complemented deleterious sites at *cis*-regulatory regions in maize hybrids. **(B)** Correlation of complemented deleterious sites at *cis*-regulatory regions and hybrid grain yield. **(C)** Correlation of complemented deleterious sites at *cis*-regulatory regions and heterosis of hybrid grain yield. **(D)** Historical changes of complemented deleterious sites at CDS regions in maize hybrids. **(E)** Correlation of complemented deleterious sites at CDS regions and hybrid grain yield. **(F)** Correlation of complemented deleterious sites at CDS regions and heterosis of grain yield.


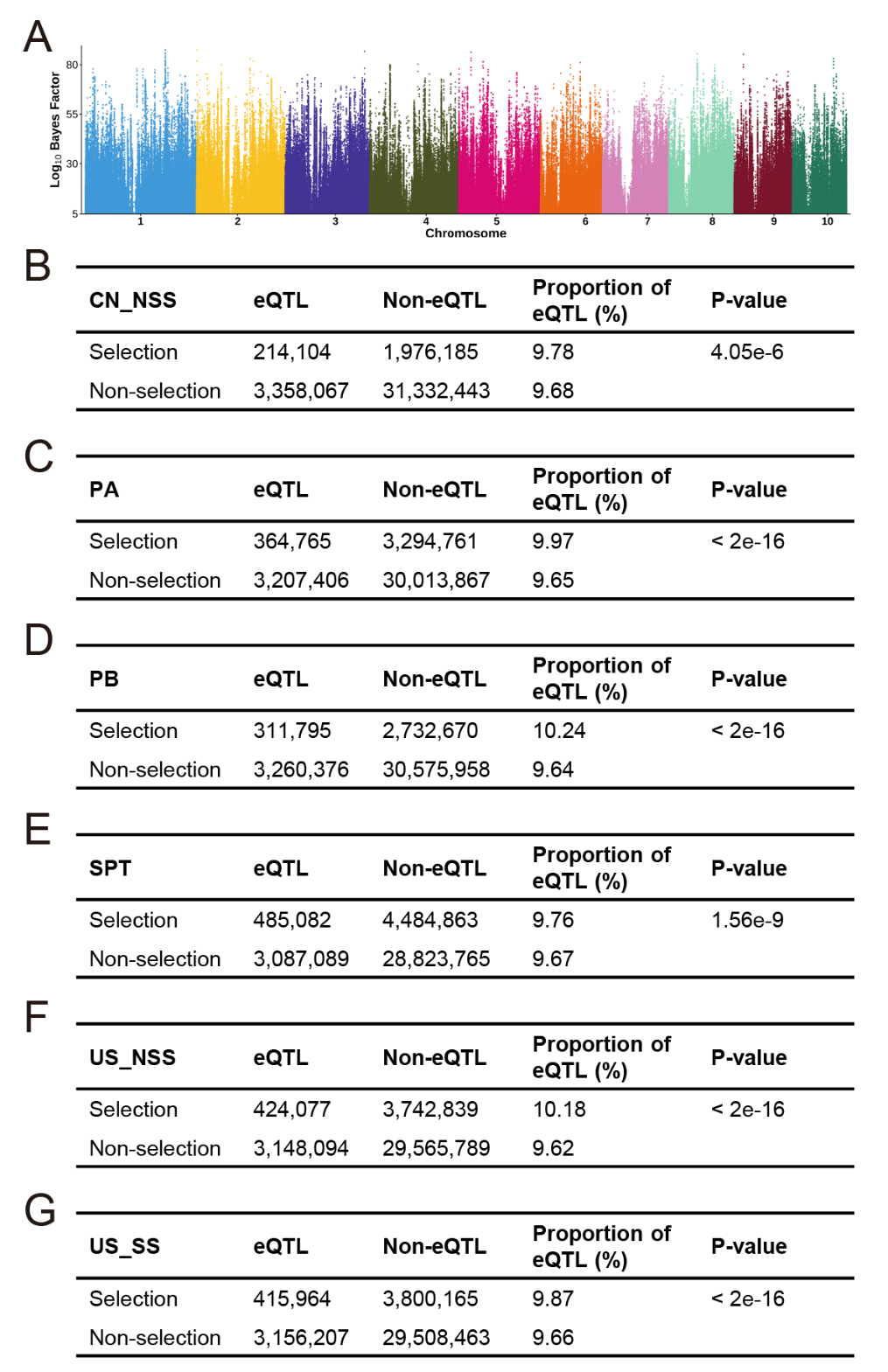


**Figure S14. Comparison of cis-eQTN and breeding selection signatures in maize. (A)** Manhattan plot of 3,572,171 *cis*-eQTNs associated with 19,208 genes. **(B-G)** Fisher’s Exact Test of the *cis*-eQTNs and breeding selection signatures in different heterotic groups (CN_NSS, PA, PB, SPT, US_NSS and US_SS) obtained by XP-CLR analysis in our previous study (Li et al., 2022).


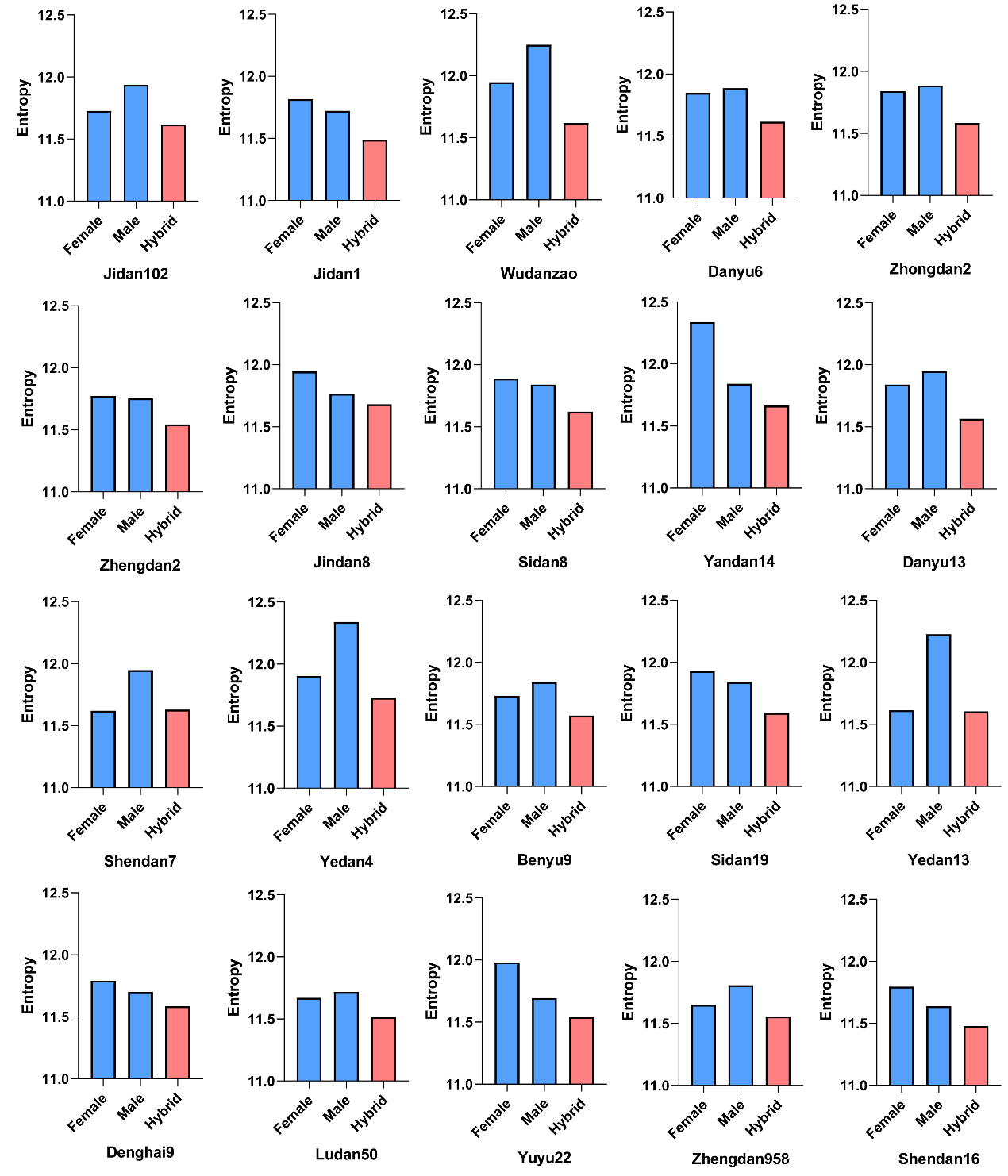


**Figure S15. Transcriptomic dynamics in maize hybrids and parental inbreds.** The Transcriptomic dynamics of 20 maize hybrids and corresponding female and male parental inbreds were calculated based on Shannon’s Entropy Model.


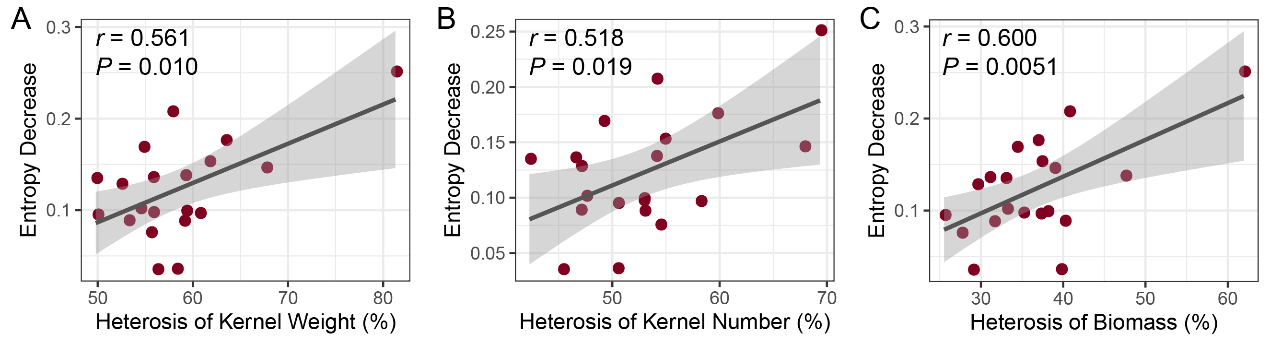


**Figure S16.** **Correlation between transcriptomic entropy reduction and phenotypic variation in hybrids. (A-C)** Correlation analysis of transcriptomic entropy reduction towards heterosis of kernel weight per ear, kernel number per ear and biomass, respectively.


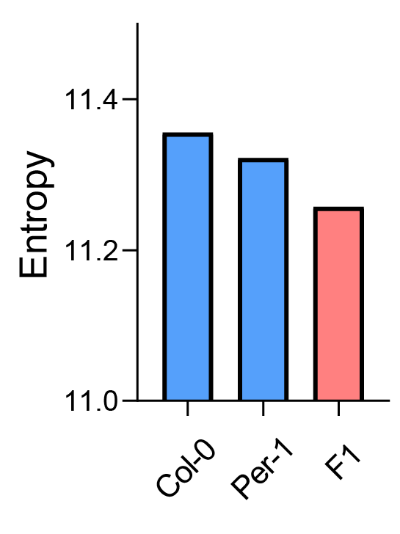


**Figure S17.** **Transcriptomic entropy of *Arabidopsis* ecotype Col-0, Per-1 and their F_1_ hybrid. (A-C)** The transcriptomic entropy in *Arabidopsis* hybrid decreased as compared to its parental ecotypes. The gene expression profiles were obtained from (Liu et al. 2021).


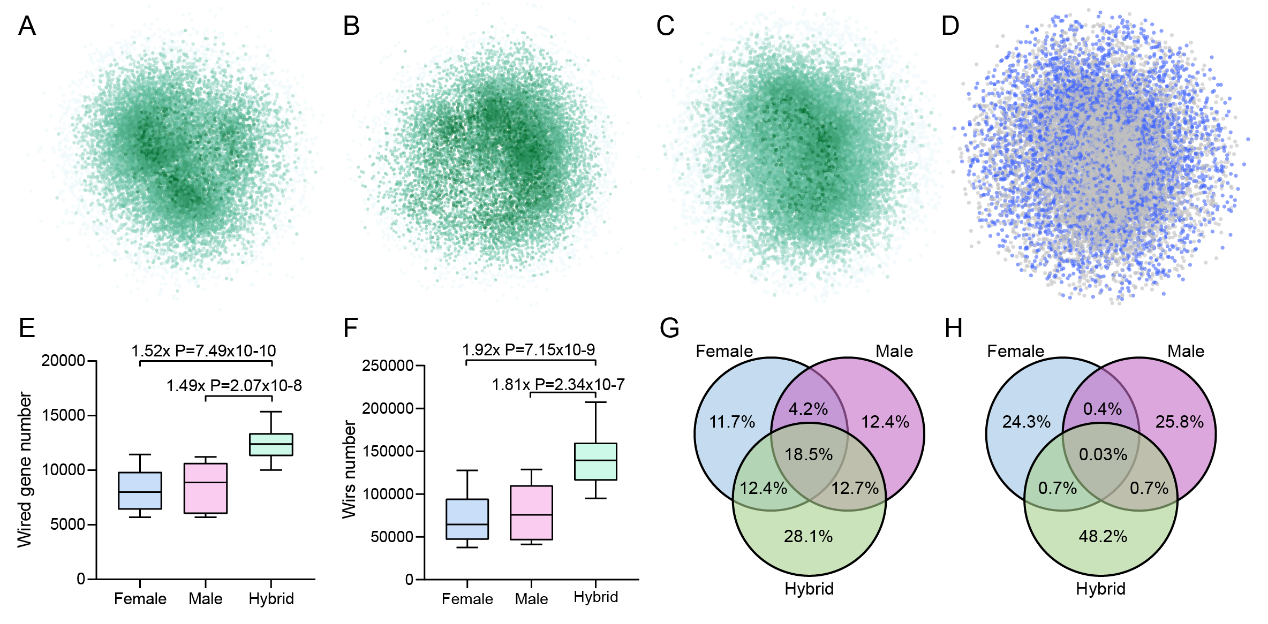


**Figure S18. Expanded gene regulatory networks in maize hybrids. (A-C)** The GRNs of maize inbred Zheng58 (A), Chang7-2 (B) and their hybrid Zhengdan958 (C) are shown. Dots represent genes in GRN and green color depth of dots summarize the number of links. Edges are omitted for clear illustration. **(D)** Comparative analysis of GRNs of Zhengdan958 and the parents Zheng58 and Chang7-2. The blue dots present the linked genes that were not detected in parental inbreds Zheng58 and Chang7-2. And the grey dots present the linked genes shared by both hybrid and parental inbreds. **(E)** Statistics of wired gene number in GRNs of maize hybrids and parental inbreds. **(F)** Statistics of wires number in GRNs of maize hybrids and parental inbreds. **(G)** Similarity and diversity of wired genes in GRNs of maize hybrids and parental inbreds. **(H)** Similarity and diversity of wires in GRNs of maize hybrids and parental inbreds.


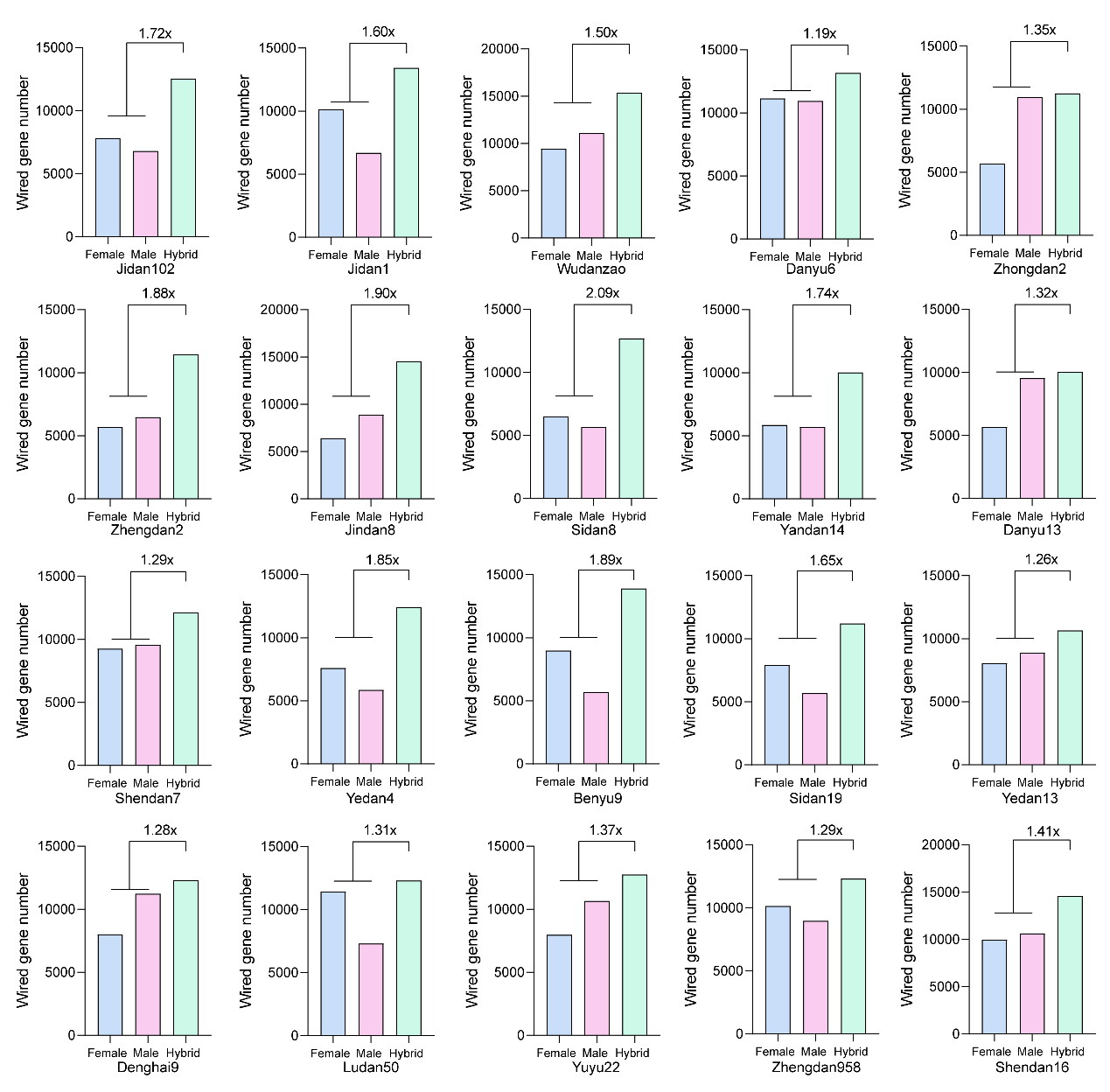


**Figure S19.** **Wired gene number in GRNs of maize hybrids and parental inbreds.** Hybrids GRNs had 1.19- to 2.09-fold number of wired genes than parental inbreds.


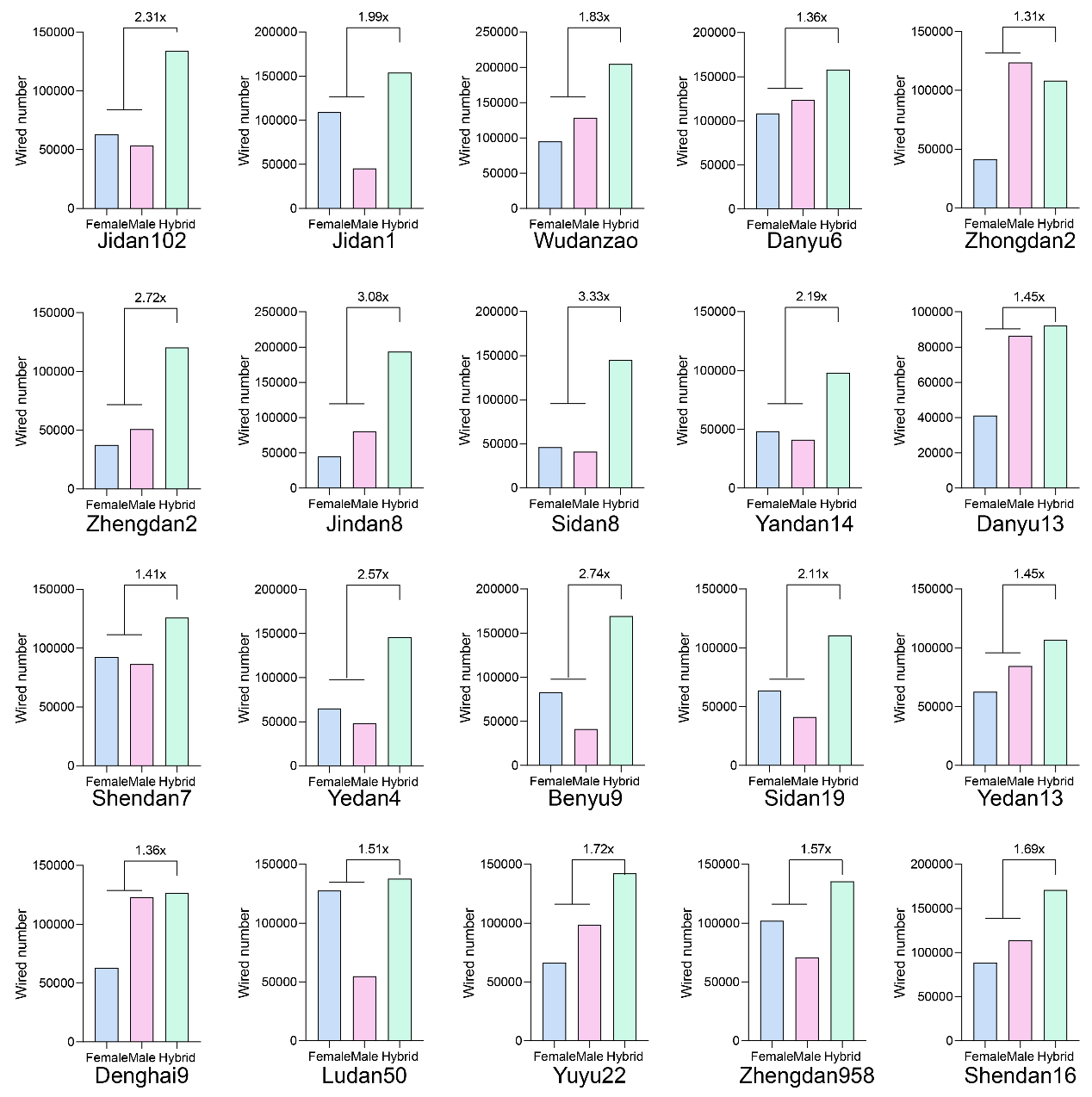


**Figure S20.** **Wires in GRNs of maize hybrids and parental inbreds.** The wires in GRNs of hybrids were 1.31- to 3.33-fold of the parental inbreds.


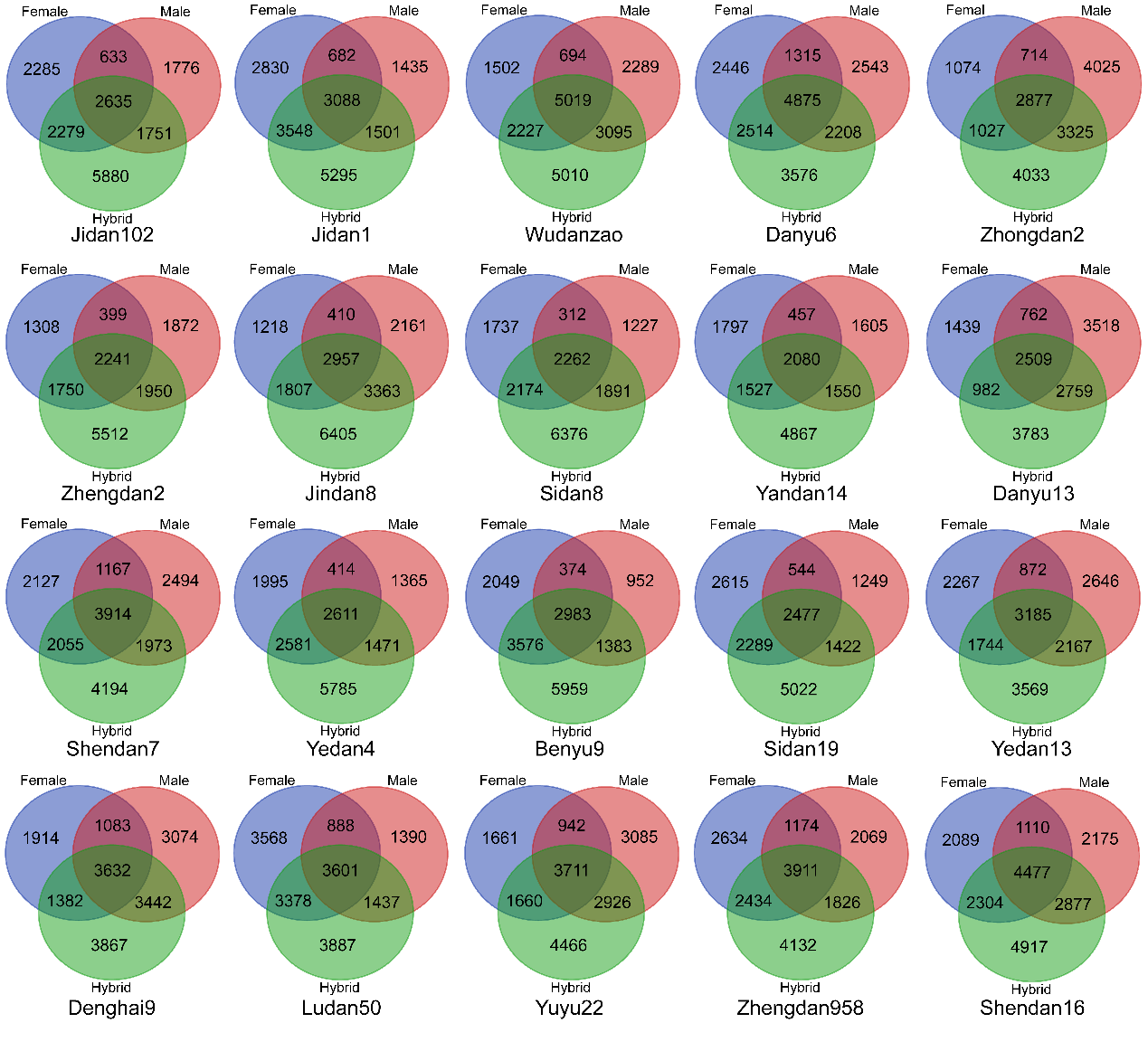


**Figure S21. Similarity and diversity of wired genes in GRNs of maize hybrids and parental inbreds.** The shared and diverse wire genes in GRNs of maize hybrids and parental inbreds are shown by Venn plot.


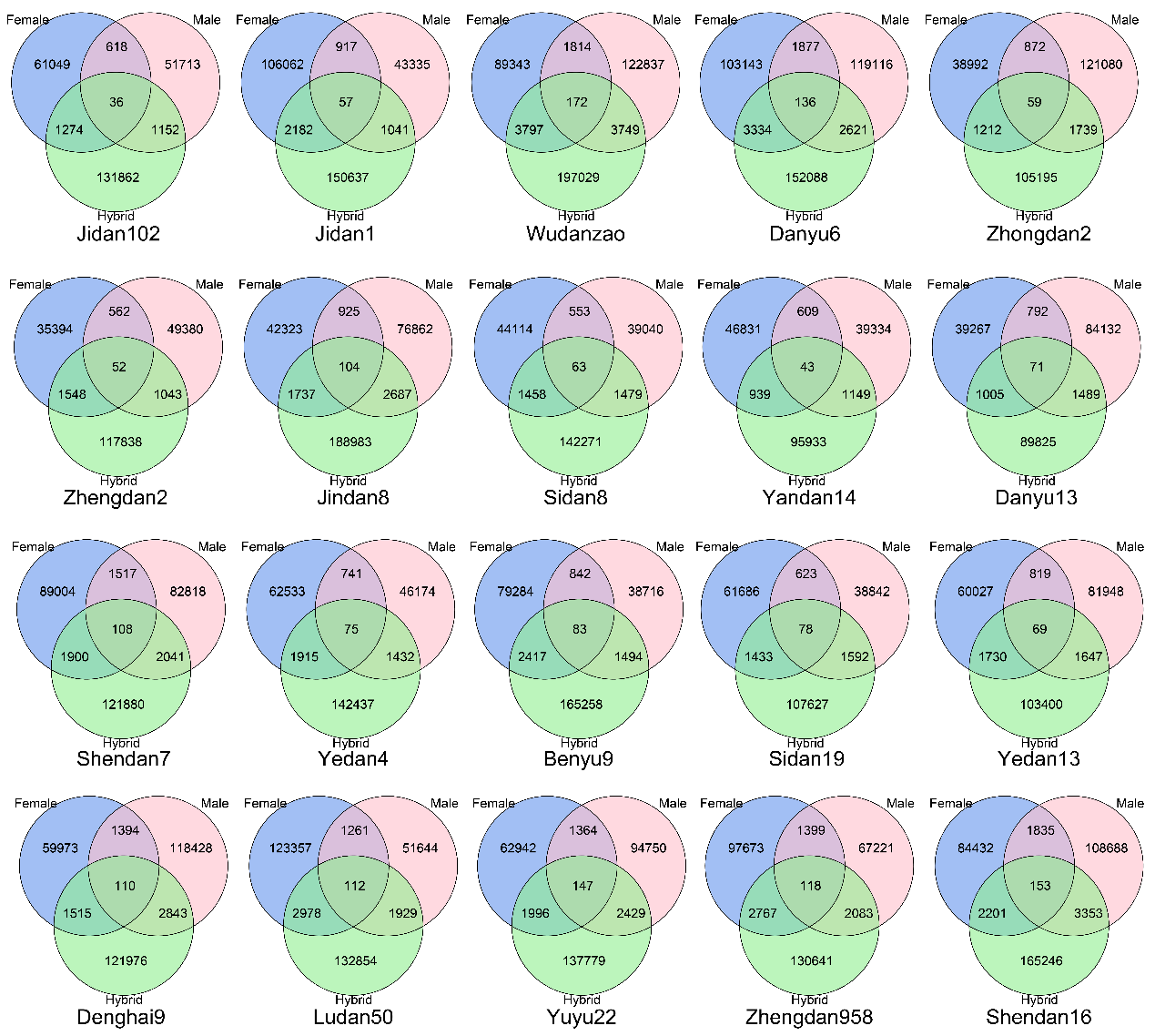


**Figure S22.** **Similarity and diversity of wires in GRNs of maize hybrids and parental inbreds.** The shared and diverse wires in GRNs of maize hybrids and parental inbreds are shown by Venn plot.
